# NSUN2-mediated m5C modification of SOCS3 mRNA modulates macrophage polarization in bladder cancer

**DOI:** 10.1038/s41419-025-08306-4

**Published:** 2025-12-07

**Authors:** Yi Tang, Xinpei Deng, Yanjun Wang, Qianghua Zhou, Chichen Zhang, Zhicheng Liu, Runhao Zheng, Jiamin Zeng, Xingliang Tan, Zhiming Wu, Kai Yao, Gangjun Yuan

**Affiliations:** 1 Department of Urology, Sun Yat-sen University Cancer Center, Guangzhou, China; 2 State Key Laboratory of Oncology in Southern China, Guangzhou, China; 3 Guangdong Provincial Clinical Research Center for Cancer, Guangzhou, China; 4 Department of Urology Oncological Surgery, Chongqing University Cancer Hospital, Chongqing, China; 5 Chongqing Key Laboratory of Translational Research for Cancer Metastasis and Individualized Treatment, Chongqing University Cancer Hospital, Chongqing, China

**Keywords:** Cancer microenvironment, Cancer immunotherapy

## Abstract

Tumor-associated macrophages (TAMs) are pivotal in facilitating the progression of cancer cells. M1 and M2 are two polarization states of TAMs with opposite functions in tumor progression. While the regulatory role of N6-adenosine (m6A) methylation in macrophage polarization has been established, the function of 5-methylcytosine (m5C) remains unclear. The presence of M2 macrophages in bladder cancer and adjacent normal tissues was validated using fluorescence-activated cell sorting (FACS) and immunofluorescence (IF). The expression of reported m5C regulators (writers, readers, erasers) was screened in M2 macrophages to identify the most relevant regulators. Mechanistic insights into how m5C methylation regulates macrophage polarization were gained through RNA immunoprecipitation (RIP) and quantitative PCR. The FACS and IF results revealed that the main active state of the tumor microenvironment (TME) was the M2 subtype in bladder cancer. Next, NOP2/Sun RNA methyltransferase family member 2 (NSUN2) was identified as the most upregulated RNA m5C methylase in M2 via a qPCR assay. According to whole-transcriptome resequencing in si-NSUN2 RAW/THP-1 cells and GO analysis, SOCS3 was determined to be downstream of NSUN2. By methylating particular sites in SOCS3 mRNA, NSUN2 inhibits both the stability and nuclear export of SOCS3 mRNA, which subsequently activates the JAK2/STAT3 signaling pathway and then promotes macrophage polarization to the M2 phenotype while inhibiting M1 polarization. Additionally, this process involves the assistance and balance of the reader YBX1 and the eraser TET2. NSUN2 methylates SOCS3 mRNA to inhibit its stability and nuclear export, which consequently promotes macrophage polarization to M2.

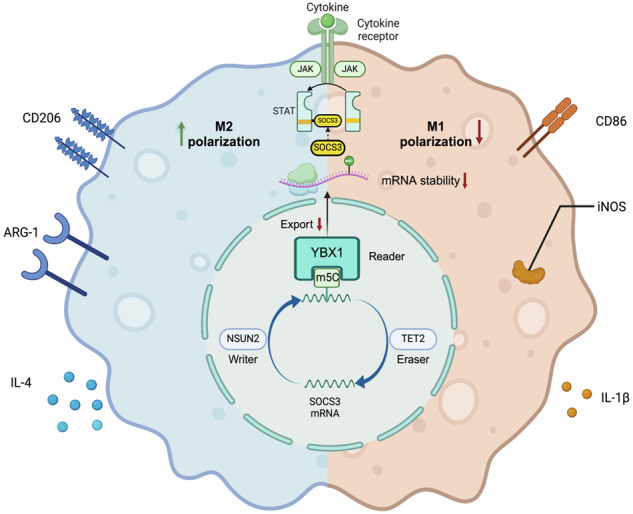

## Background

Tumor-associated macrophages (TAMs) are pivotal in facilitating the progression of various types of solid tumors [1, 2]. TAMs can be divided into two subgroups characterized according to their active state: M1 and M2 [3, 4]. M1 macrophages have proinflammatory and antitumor activities, whereas M2 macrophages have the opposite functions [5]. Additionally, according to Wang et al., the polarization state of TAMs is essential for the prognosis of bladder cancer (BC) patients, and M2 TAMs are associated with worse outcomes and clinical stages [6]. However, the state between M1 and M2 is not stable and is subject to reversal. For example, Bossche et al. revealed that M2 macrophages are highly plastic and can be repolarized into the M1 state by the addition of LPS and IFNγ [4]. Additionally, Wang et al. explored the reprogramming of M2-like TAMs into antitumor M1-like TAMs as a novel cancer treatment [7].

The process of macrophage polarization is complicated, and RNA epigenetic modification has been shown to play a critical role in this regulation [8, 9]. Du et al. reported that METTL14-regulated macrophage activation via N6-adenosine methylation (m6A) of SOCS1 mRNA was required to maintain the negative feedback control of macrophage activation in response to bacterial infection [10]. Similarly, according to Ma et al., YTHDF, an m6A reader, has been shown to mediate the reprogramming of TAMs and control their antitumor immunity through CD8 + T cells [11].

5-Methylcytosine methylation (m5C) is another important form of RNA methylation, and its role in RNA metabolic regulation of cancer development has been described [12, 13]. Xu et al. reported that mRNA m5C methylation is upregulated in gastric cancer and promotes its progression [14]. Previous reports have identified a total of 17 enzymes associated with the regulation of m5C RNA methylation [15]. However, it remains unclear which specific enzyme is involved in the regulation of TAMs in bladder cancer, and further research is needed.

Thus, in this study, we investigated the role of NSUN2 in the process of macrophage polarization in BC and showed that NSUN2 regulated macrophage polarization via m5C mRNA modification to control both the stability and nuclear export ability of SOCS3 mRNA. Additionally, this process requires the assistance and balance of the reader Y-box binding protein 1 (YBX1) and the eraser TET2.

## Methods

### Patient cohort, tissue sample, and research ethics

All BCa samples were obtained from patients diagnosed with BCa who had undergone surgery at Sun Yat-sen University Cancer Center. According to the TNM Staging System for Bladder Cancer (8th edition, 2017), data on each patient’s clinical and pathological information, as well as their survival, were compiled. This study was approved by the SYSUCC Ethics Committee (B2024-058-01), and informed consent was acquired.

### Immunofluorescence (IF) assay

The expression of NSUN2 in macrophages in BCa patients was determined by IF using purified polyclonal antibodies (Details of the used antibodies is listed in Table S1). Initially, 4 µm paraffin-embedded tissue sections from 64 patients were subjected to deparaffinization using xylene, followed by rehydration through a graded alcohol series. Citrate buffer was used in antigen retrieval 15 minutes, and nonspecific antigens were blocked with 5% goat serum for 30 min at 37 degrees. Then, the cells were incubated with NSUN2 and CD68 antibodies at 4 degrees overnight and then the cells were incubated with DyLight 488 and DyLight 594 goat anti-rabbit/mouse secondary antibodies. Finally, the cells were incubated with DAPI for 5 min at 37 degrees.

### Cell lines, culture conditions, and transfection

The RAW and THP-1 macrophage lines were purchased from the Type Culture Collection of the Chinese Academy of Sciences (Shanghai, China). To obtain bone marrow-derived macrophages (BMDMs), we flushed mouse bone marrow from tibias and femurs, and then 20 ng/ml M-CSF was added to induce the differentiation of bone marrow cells for 1 week [16]. For peripheral blood mononuclear cells (PBMCs), we isolated mononuclear cells (MNCs) from whole blood with a SepMate™ (STEMCELL, #86450). CD14 + CD16- monocytes were subsequently isolated with an EasySep™ Human Monocyte Isolation Kit (STEMCELL, #19359). Finally, 20 ng/ml CSF-1 was added to monocytes to obtain differentiated macrophages [17]. These cell lines were maintained in Dulbecco’s modified Eagle’s medium (DMEM) supplemented with 10% fetal bovine serum (FBS; Gibco, Waltham, MA, USA), and the cells were cultured at 37 °C in a 5% CO2 incubator. To silence NSUN2, small interfering RNAs (siRNAs) were designed and synthesized by GenePharma (Shanghai, China) and transfected with Lipo8000TM Transfection Reagent (Beyotime). The effective sequences are shown in Table S3.

### Western blot (WB)

The cells were treated with RIPA lysis buffer (Beyotime) together with 1% phosphatase and protease inhibitors to extract the proteins, after which the proteins were separated by 10% SDS‒PAGE (EpiZyme, Cambridge, MA, USA). Then, the proteins were transferred onto PVDF membranes (Pierce Biotechnology, Waltham, MA, USA), which were blocked with 5% milk at 37 °C for 1 h. These membranes were incubated with primary antibodies at 4 °C overnight. The membranes were subsequently incubated with secondary antibodies for 2 hours. Finally, the membranes were exposed to enhanced chemiluminescence (ECL) reagents (Abcam, Cambridge, UK). The antibodies used are listed in Table S1.

### Quantitative real-time polymerase chain reaction (qPCR)

RNA was extracted with TRIzol and then reverse transcribed (HiScript Q RT SuperMix Kit, Vazyme, Nanjing, China) and amplified (ChamQ SYBR qPCR Green Master Mix Kit, Vazyme) according to the manufacturer’s directions. The relative expression of the target genes was calculated by the 2(−ΔΔCt) method and normalized against the expression of GAPDH. The primers used are listed in Table S2.

### M0, M1/M2 macrophage induction of different cell lines

M0 macrophage induction of PBMCs/BMDMs was performed as described above (RAW cells were already M0 macrophages). For M0 induction, THP-1 cells were exposed to 100 ng/ml PMA for 24 hours. For M1 induction of M0 macrophages, 20 ng/ml LPS was added for 48 hours. For M2 induction, we added 20 ng/ml IL-4 and IL-13 to M0 cells for 48 hours.

### ELISA analysis of supernatants from macrophages after exosome treatment

The levels of IL-10, TGFβ, and CXCL1 in the cell culture medium were determined using ELISA with a Quantikine human ELISA kit from Wuhan Fine Biotech Co., Ltd., following the manufacturer’s guidelines.

### Coculture of macrophages treated with exosomes with BCa cells

To induce differentiation into M0 macrophages, THP-1 cells were seeded in a 24-well plate and exposed to PMA. After 12 hours, the culture medium was exchanged for a new medium, and a small chamber with BCa cells was placed on top of the 24-well plate. The upper chamber had medium without serum, whereas the lower chamber was filled with medium that included 10% serum. After 24 hours of coculture, the cells were fixed with paraformaldehyde, stained with crystal violet, and photographed.

### Removing macrophages in C57 mice with clodronate-liposome

Injecting 10 mg/kg clodronate-liposome according to body weight into C57 mice by tail vein every week. To validate the effect of macrophage depletion, mice were sacrificed 3 days after the injection, and single-cell suspensions from the spleen were obtained for flow cytometry that analyze the proportion of F4/80^+^ cells in the C57 spleen. The animal experiments were approved by the SYSUCC Animal Ethics Committee (L102022023009K).

### RNA pull-down assay

RIPA cell lysis buffer was used to prepare the cell lysates. In brief, 50 µl of streptavidin magnetic beads were combined with 50 pmol of 3’-biotin-labeled RNA probes and agitated for 30 minutes. Following incubation, 100 mg of protein lysate was added, and the mixture was agitated for 60 minutes at 4 °C.

### RIP assay

An EZ-Magna RIP kit (NO. 17-701) was used. Briefly, the cells were lysed on ice with RIP lysis buffer. Then, 50 µl of protein A/G magnetic beads, 100 µl of cell lysate and 900 µl of RIP buffer were added to a clean microtube and rotated at 4 °C for 3 h. Ten microliters of cell lysate was kept as input and stored at −80 °C. Finally, the RNA was purified, collected, and converted to cDNA for qPCR.

### mRNA stability assay

Each sample was harvested at 0, 3, 6, and 7 hours after treatment with actinomycin D (2 µM). Total RNA was isolated and converted to cDNA for RT‒qPCR analysis.

### Statistical analysis

Statistical analysis was performed by SPSS software (version 25.0). All the data are presented as the means ± SDs. Student’s *t*-test or one-way ANOVA was performed to analyze the differences between the two groups. Benjamini-Hochberg adjustment was used to correct the false discovery rates in multiple comparisons. Survival analysis was performed with Kaplan–Meier survival curves. The associations were analyzed by Pearson’s chi-square test. A *p*-value < 0.05 was considered significant.

## Results

### Infiltration of TAMs in BC tissues

**Fig. 1:**
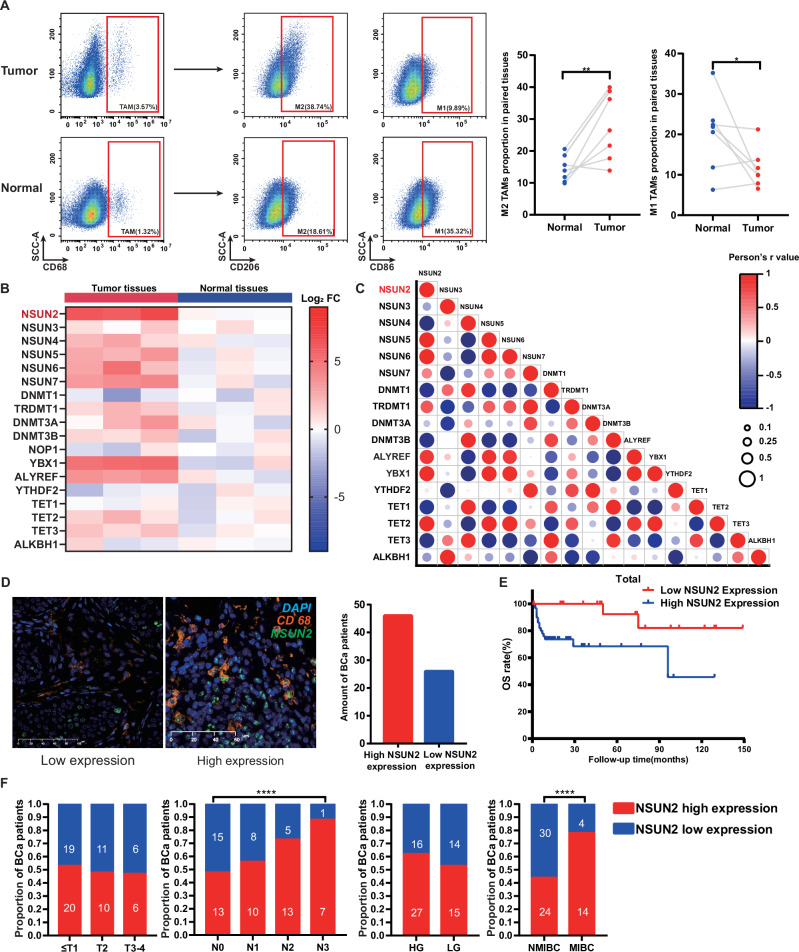
M2 polarization is increased in BCa tumor-surrounding tissues, and NSUN2 is increased in TAMs. **A** BCa tissues tend to have a greater M2 proportion and a lower M1 proportion than normal tissues do. **B** A series of m5C writers are upregulated in BCa TAMs, and NSUN2 is the most highly expressed writer among these m5C writers. **C** Correlation analysis of m5C-related genes revealed that NSUN2 expression is related to various m5C readers and erasers, such as ALYREF, YBX1, and TET2. **D** IF was performed to evaluate NSUN2 expression in BCa TAMs, and patterns of low expression and high expression are shown. **E** Patients with high NSUN2 levels tend to have poorer clinical outcomes. **F** High NSUN2 expression in BCa TAMs is associated with N stage and muscle invasion. *p < 0.05; **p < 0.01; ***p < 0.001; ****p < 0.0001. Statistics are presented as the means ± SDs of three independent experiments.

To validate the distribution of M1/M2 TAMs in BC tissues, fluorescence-activated cell sorting (FACS) was used in 6 pairs of tumor and corresponding normal tissues. The results revealed that CD68^+^ macrophages were more likely to be present in surrounding tumor-surrounding tissues than in normal tissues. Among the population of CD68^+^ cells, CD206^+^CD68^+^ (M2) and CD86^+^CD68^+^ (M1) cells accounted for 36.2% and 12.7%, respectively, of the population in tumor-surrounding tissues, whereas they accounted for 13.2% (M2) and 45.3% (M1) of the population in normal tissues (Fig. 1A). These results revealed that M2 macrophages were the main subtype of TAMs in BC tumor-surrounding tissues.

### NSUN2 expression in TAMs and its clinical association with BC patients

We wondered whether m5C RNA methylases play a role in the regulation of M1 and M2 distribution. According to Ma et al., a total of ten m5C RNA methylases have been identified since then. Thus, a quantitative PCR (qPCR) assay was performed to compare the expression of these m5C RNA methylases in tumor-surrounding and normal tissues. As shown in Fig. 1B, several kinds of mRNAs were highly expressed in tumor-surrounding tissues, among which NSUN2 was the most significantly elevated. To validate the effect of NSUN2 on macrophages, several cell lines derived from both human and mouse mononuclear leukocytes (THP-1 cells, RAW cells) were used to construct macrophage models stimulated with IL-4. Whole-transcriptome resequencing was subsequently performed in these stimulated cells, which revealed that NSUN2 was commonly upregulated in M2 macrophages, and GO analysis showed that NSUN2-related signaling pathways, such as regulation of mRNA stability or RNA transport, were enriched, which indicated the potential of NSUN2 in M2 polarization via m5C methylation (Fig. S1).

NSUN2 is the most common m5C RNA methylase in BCa, which is also highly expressed in cancer cell, and its role in BCa cell has been revealed. We explored the role of NSUN2 in the clinical outcome of bladder cancer by analyzing relevant data from public databases. Results showed that high NSUN2 expression is associated with a poor response and prognosis to immunotherapy such as anti-CTLA-4 and anti PD-1/PD-L1 therapy (Fig. S2). However, all current BCa studies examined the NSUN2 expression in whole tumor tissue, including tumor cells and immune cells. Therefore, we additionally analyzed the associations of NSUN2 expression in TAMs with the clinical characteristics of BCa patients. The results revealed that patients with high NSUN2-expressing TAMs had shorter 5-year overall survival than patients with low NSUN2-expressing TAMs did (Fig. 1E). In addition, high numbers of NSUN2-expressing TAMs seemed to be related to lymph node metastasis and muscle invasion in patients (Fig. 1F). Taken together, these results indicate that high NSUN2 expression in TAMs is associated with a poor prognosis in BC patients.

### NSUN2 regulates the polarization and function of macrophages

**Fig. 2:**
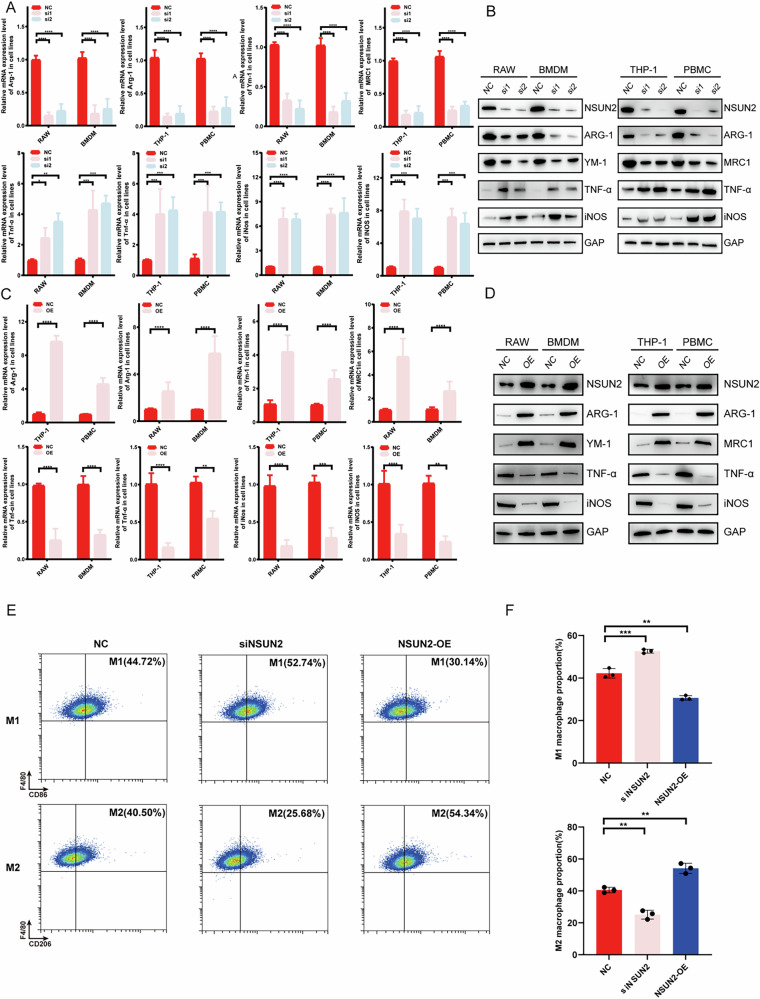
Expression of NSUN2 changes the balance of M1/M2 polarization. **A**, **B** Knockdown of NSUN2 promoted M1 polarization but inhibited M2 polarization in macrophages. **C**, **D** Overexpression of NSUN2 promoted M2 polarization but inhibited M1 polarization of macrophages. **E**, **F** Flow cytometry of RAW revealed a greater proportion of M1 and a lower proportion of M2 in the siNSUN2 group and a lower M1 proportion and higher M2 proportion in the NSUN2-OE group. (Flow cytometry result of THP-1 was showed in Fig S3). *p < 0.05; **p < 0.01; ***p < 0.001; ****p < 0.0001. Statistics are presented as the means ± SDs of three independent experiments.

To further validate the role of NSUN2 in M2 polarization, siRNA technology was used to knock down NSUN2 expression in four mononuclear leukocytes (RAW, THP-1, BMDM and PBMC). As expected, repression of NSUN2 expression increased both the mRNA and protein expression levels of M1-related genes, such as IL-10 and Arg-1, but reduced the mRNA and protein expression levels of M2-related genes, such as TNF-α and iNOS (Fig. 2A, B). A similar trend was observed for the levels of corresponding cytokines excreted by M1/M2 macrophages (Fig. S3A). Moreover, after NSUN2 was overexpressed with a plasmid, the opposite effect on M1/M2 polarization in various macrophages was observed (Fig. 2C and D). In addition, the growth of BCa cells was greater in the NSUN2-knockdown M2 macrophages than in the control cells (Fig. S3), indicating that the knockdown of NSUN2 inhibited the protumor effect of M2 macrophages on BCa cells. In the flow cytometry, CD 86^+^/F4/80^+^(or CD68^+^ in human cells) cells were determined as M1 macrophage, and CD 206^+^/F4/80^+^(or CD68^+^ in human cells) cells were determined as M2 macrophage. Results of these flow cytometry in RAW and THP-1 cells also showed that knockdown of NSUN2 decreased the M2 proportion while increasing M1 proportion, and over-expression of NSUN2 showed the opposite result.(Figs. 2E, F and S3). Taken together, these results indicate that NSUN2 plays a critical role in regulating the process of macrophage polarization and its active function.

#### Knocking down NSUN2 increased the expression of SOCS3, and NSUN2 mediates the polarization of macrophages via the SOCS3/JAK2/STAT3 pathway

**Fig. 3:**
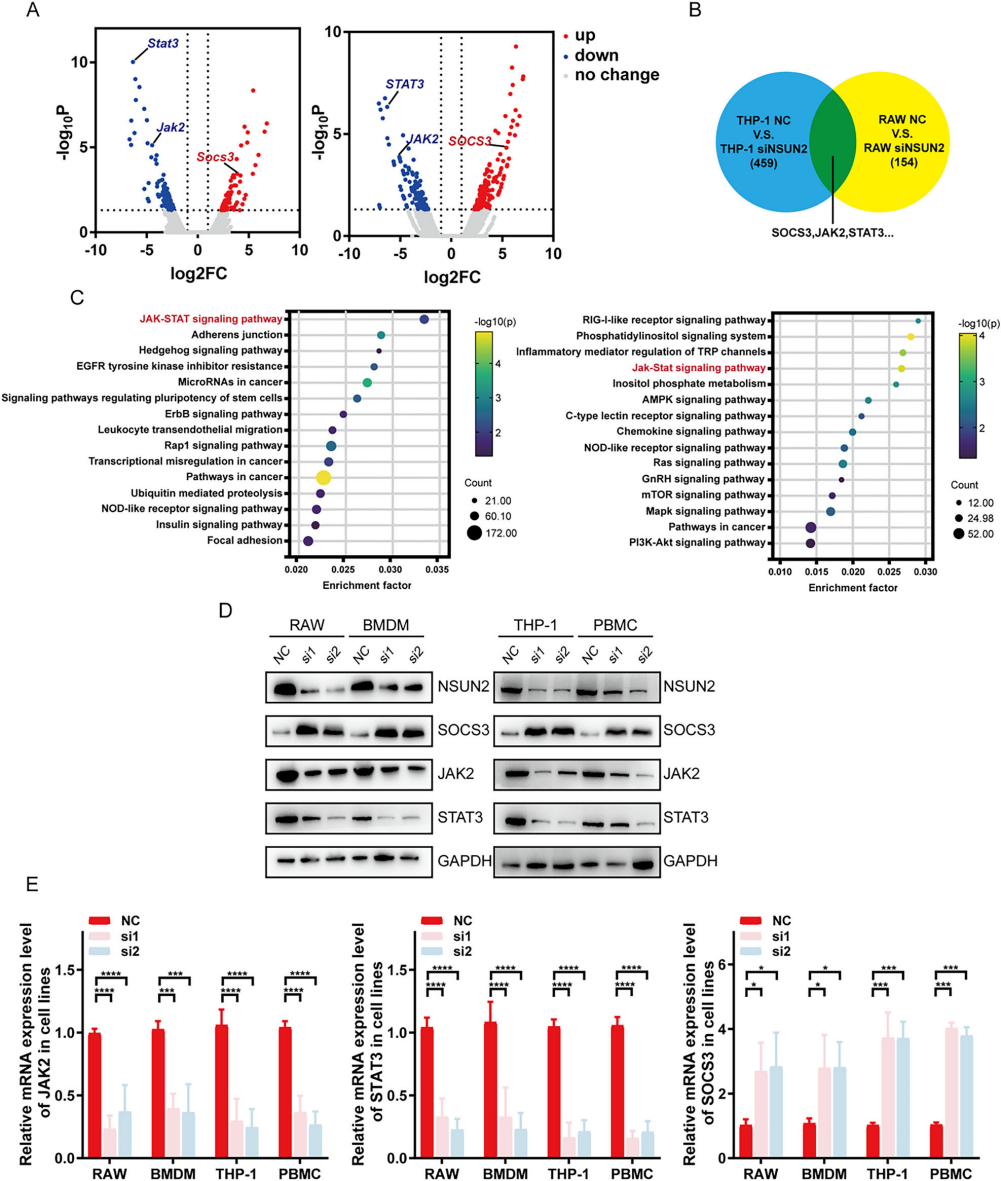
The SOCS3/JAK2/STAT3 pathway is the downstream pathway of NSUN2. **A** JAK2/STAT3 were downregulated, whereas SOCS3 was upregulated, in the siNSUN2 group. **B** SOCS3/JAK2/STAT3 expression was altered in both RAW264.7 and THP-1 cells. **C** KEGG pathway results revealed that JAK2/STAT3 were enriched in both the siNSUN2 group of RAW264.7 cells and the control group of THP-1 cells. **D**, **E** WB and PCR results confirmed that NSUN2 knockdown upregulated SOCS3 and downregulated JAK2/STAT3. *p < 0.05; **p < 0.01; ***p < 0.001; ****p < 0.0001. Statistics are presented as the means ± SDs of three independent experiments.

To investigate the underlying mechanism by which NSUN2 regulates macrophage polarization, a whole-transcriptome resequencing assay was performed in siNSUN2 and control cells. GO analysis was subsequently used to screen for genes downstream of NSUN2. SOCS3 was the most significantly upregulated gene, as illustrated in Fig. 3A and B. Furthermore, as depicted in Fig. 3C, we focused on signaling pathways related to macrophage polarization. Consistent with these findings, the subsequent WB and qPCR results revealed that the mRNA and protein levels of SOCS3 were elevated (Fig. 3D, E). According to data from public databases, SOCS3 also play an essential role in the immunotherapy of BCa patients. And high SOCS3 expression indicates better response and prognosis to immunotherapy.

**Fig. 4:**
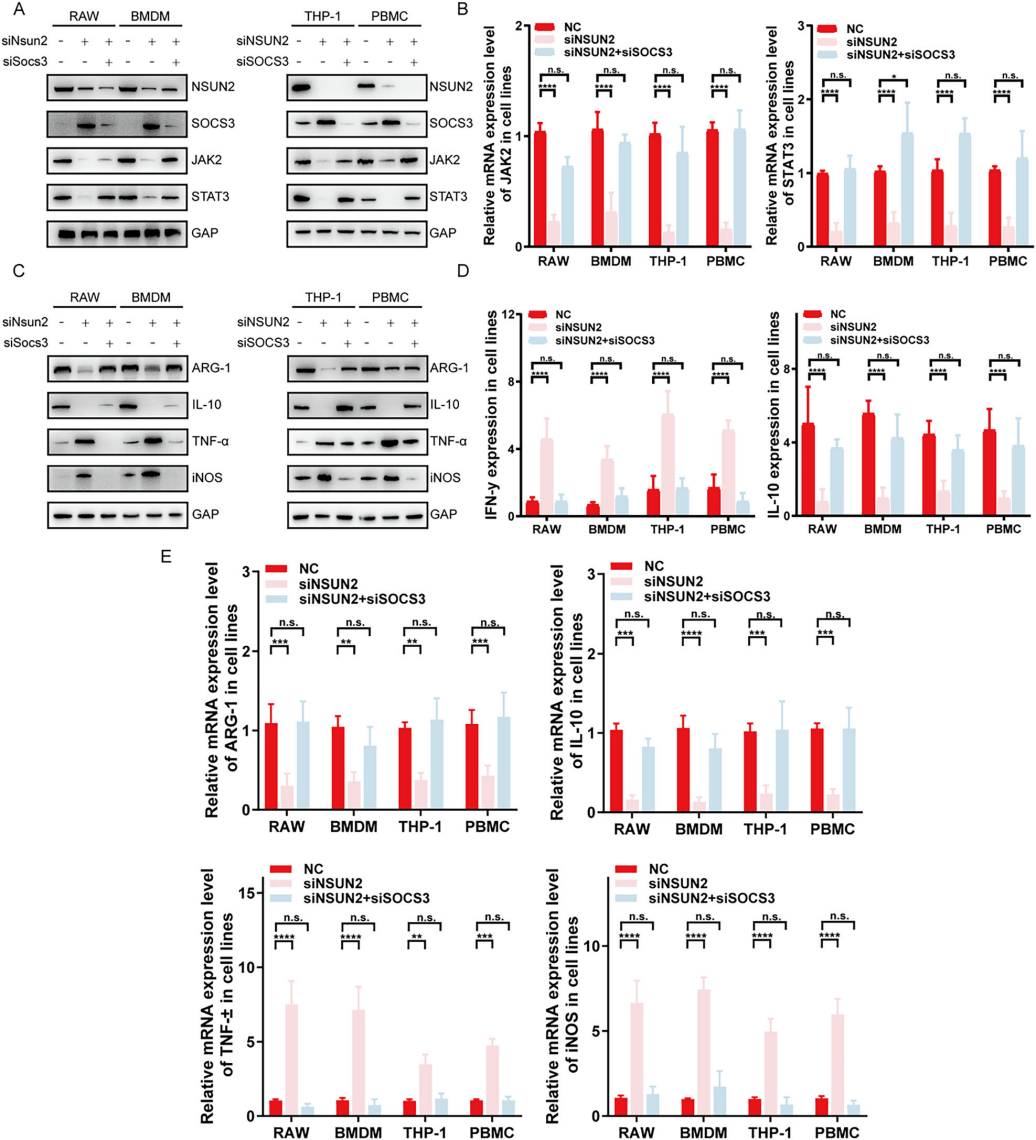
NSUN2 mediates the polarization of macrophages via the SOCS3/JAK2/STAT3 pathway. **A**, **B** The JAK2/STAT3 pathway in the siNSUN2 group was rescued after SOCS3 was also knocked down. **C**–**E** The expression of M1/M2-related mRNAs and proteins and the secretion of cytokines in the siNSUN2 group were rescued after SOCS3 was knocked down. The promotion of M1 polarization and reduction in M2 polarization were reversed in the siNSUN2-siSOCS3 group. *p < 0.05; **p < 0.01; ***p < 0.001; ****p < 0.0001. n.s. stands for non-significant. Statistics are presented as the means ± SDs of three independent experiments.

According to Rottenberg et al., the suppressor of cytokine signaling (SOCS) family is an important group of molecules that usually regulate cytokine or hormone signaling [18]. Members of the SOCS family can bind to JAK kinase, which can prevent its phosphorylation and promote its degradation [18]. Additionally, SOCS3 is known to be a negative regulator of inflammatory activities via the JAK2/STAT3 signaling pathway, and SOCS family members, such as SOCS1, were found to participate in the regulation of macrophage polarization [19–21**]**. Thus, we also detected the protein levels of JAK2/STAT3 pathway components, and the results revealed that the knockdown of NSUN2 led to a reduction in JAK2 and STAT3. Furthermore, we found that knockdown of SOCS3 in si-NSUN2 cells decreased the protein and mRNA expression of SOCS3 and rescued the levels of p-JAK2 and p-STAT3 (Fig. 3D, E). Consistent with these results, knockdown of SOCS3 remodeled the polarization and ability of macrophages caused by NSUN2 inhibition (Fig. 4A–E, S4). Taken together, these results suggested that NSUN2 promoted macrophage polarization in a manner dependent on the SOCS3-JAK2-STAT3 signaling pathway.

#### Validating that NSUN2 also mediates the polarization of TAMs via the SOCS3 pathway, both in human bladder cancer specimens and in vivo

**Fig. 5:**
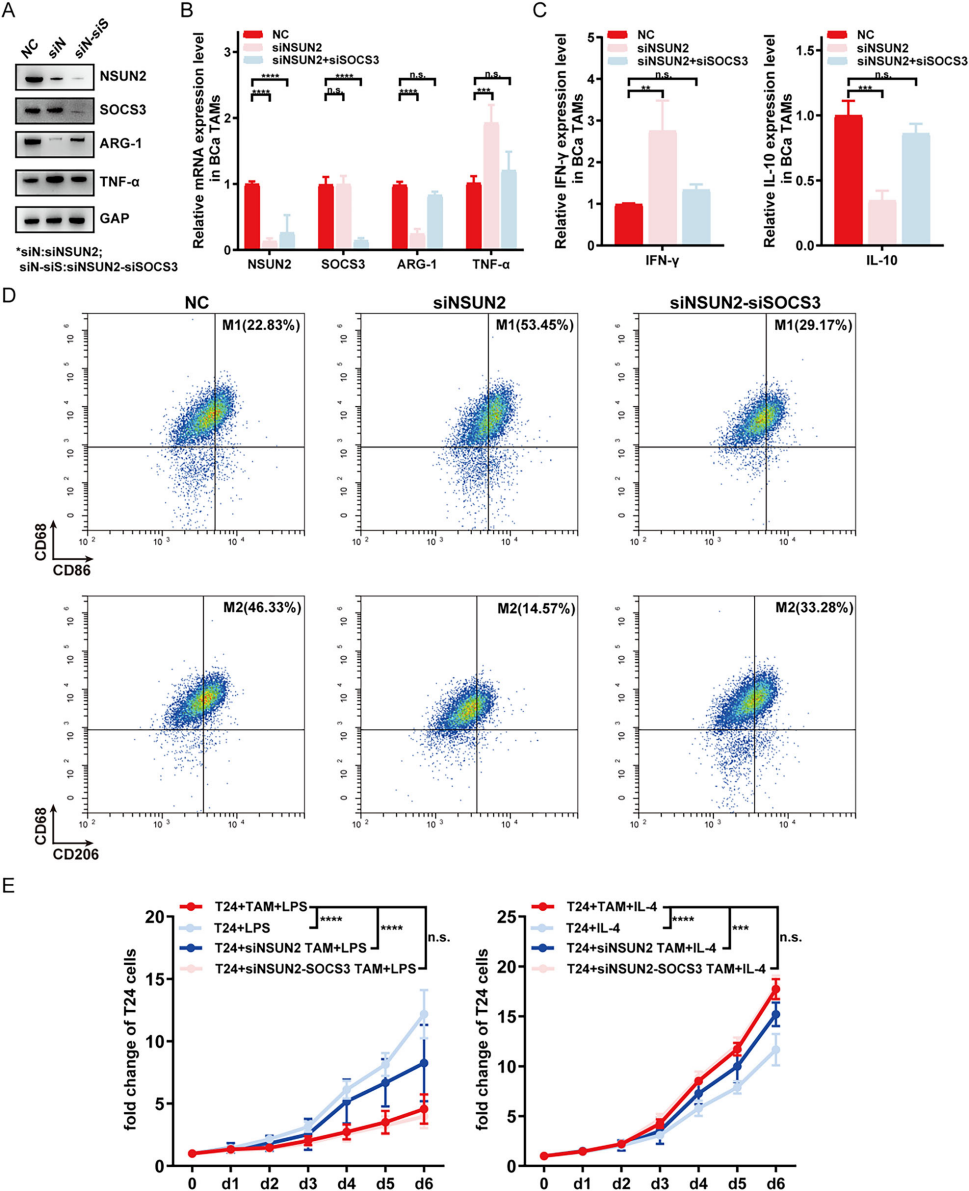
Validating role of NSUN2 and its downstream molecules SOCS3 in the polarization of TAMs in tumor specimens of Bladder cancer patients. **A**–**C** After knocking down NSUN2 in TAMs of BCa patients, both WB, PCR and ELISA showed that siNSUN2 group showed up-regulation of M1-related genes and down-regulation of M2-related genes, while the afterward knock-down of SOCS3 rescued these changes. **D** Results of flow cytometry revealed a greater proportion of M1 and a lower proportion of M2 in the siNSUN2 group while results in siNSUN2-siSOCS3 group are similar to the NC group in BCa TAMs. **E** NSUN2 promoted the pro-tumor effects of M2 TAMs while inhibiting the anti-tumor effects of M1 TAMs, and SOCS3 rescued the above effects brought by NSUN2.

Consider that all evaluated macrophage subsets above are cell lines or conditioned cells derived from bone marrow precursors rather than macrophages in human BC tissues. To validate whether NSUN2 and SOCS3 still plays a similar role in BC patients, we isolated TAMs from human bladder cancer specimens with FACS described as above. Following WB, qPCR, ELISA, and flow cytometry improved our previous theory, that is NSUN2 promoted M2 polarization and inhibited M1 polarization of TAMs in BC tissues (Fig. 5A–E). Also, knockdown of SOCS3 remodeled the polarization and ability of macrophages caused by NSUN2 inhibition.

**Fig. 6:**
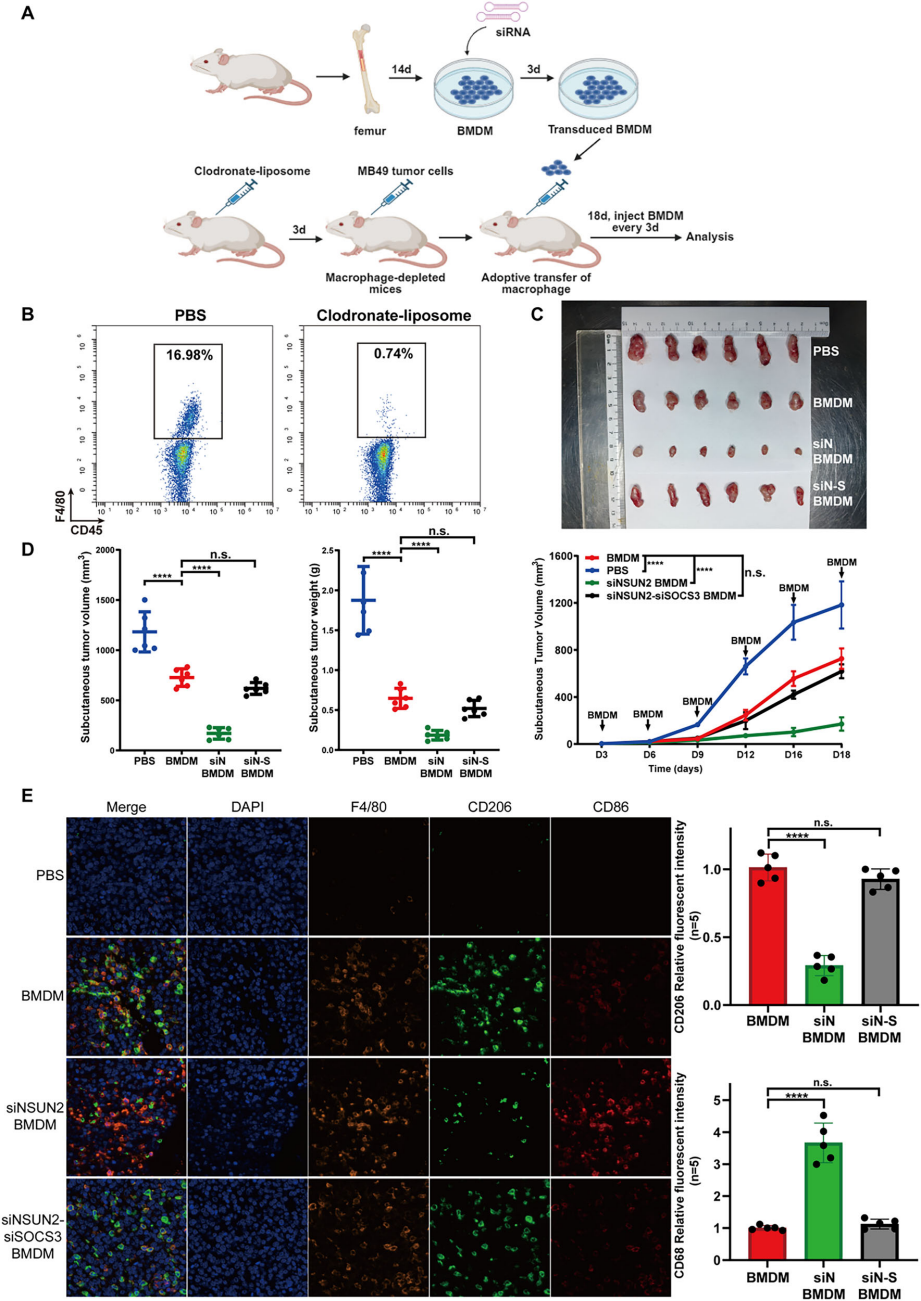
Validating role of NSUN2 and its downstream molecules SOCS3 in the polarization of TAMs in vivo. **A** After removing all macrophages in C57 with clodronate-liposome and injected with bladder cancer cells, C57 was injected with untreated BMDM or transduced BMDM (siNSUN2/siNSUN2-siSOCS3). **B** Flow cytometry was used to validate that macrophages of C57 was removed. **C**, **D** C57 injected with untreated BMDM have a smaller size of tumor compared with C57 injected with PBS. Moreover, C57 injected with siNSUN2 BMDM tend to have the smallest tumor size among 4 groups while the tumor size of siNSUN2-siSOCS3 BMDM group is similar to untreated BMDM group. **E** Results of IF showed that untreated BMDM transfer to C57 has a greater M2 proportion. However, knockdown of NSUN2 inhibited the M2 polarization and enhanced M1 polarization in vivo, while knockdown of SOCS3 in vivo rescued the above changes brought by NSUN2.

Moreover, to validate the importance of the NSUN2-centric pathway in polarization of TAMs in vivo, we removed all macrophages in C57 mice with clodronate-liposome, and then C57 mice were injected with bladder cancer cells and untreated BMDM or siNSUN2/siNSUN2-siSOCS3 BMDM to validate the function of NSUN2 and SOCS3 in vivo (Fig. 6A, B). Results showed that C57 injected with all kinds of BMDM tend to have a smaller tumor size compared with PBS. Moreover, among all BMDM subgroups, tumor size in the siNSUN2 group is smallest, and tumor size in the siNSUN2-siSOCS3 group and the NC group don’t have a significant difference, indicating that knock-down of NSUN2 inhibited tumor growth in vivo while SOCS3 rescued this inhibition (Fig. 6C, D). Also, results of IF showed that knockdown of NSUN2 inhibited the M2 polarization and enhanced M1 polarization in vivo, while knockdown of SOCS3 in vivo rescued the above changes brought by NSUN2 (Fig. 6E).

### NSUN2 methylated SOCS3 mRNA

**Fig. 7:**
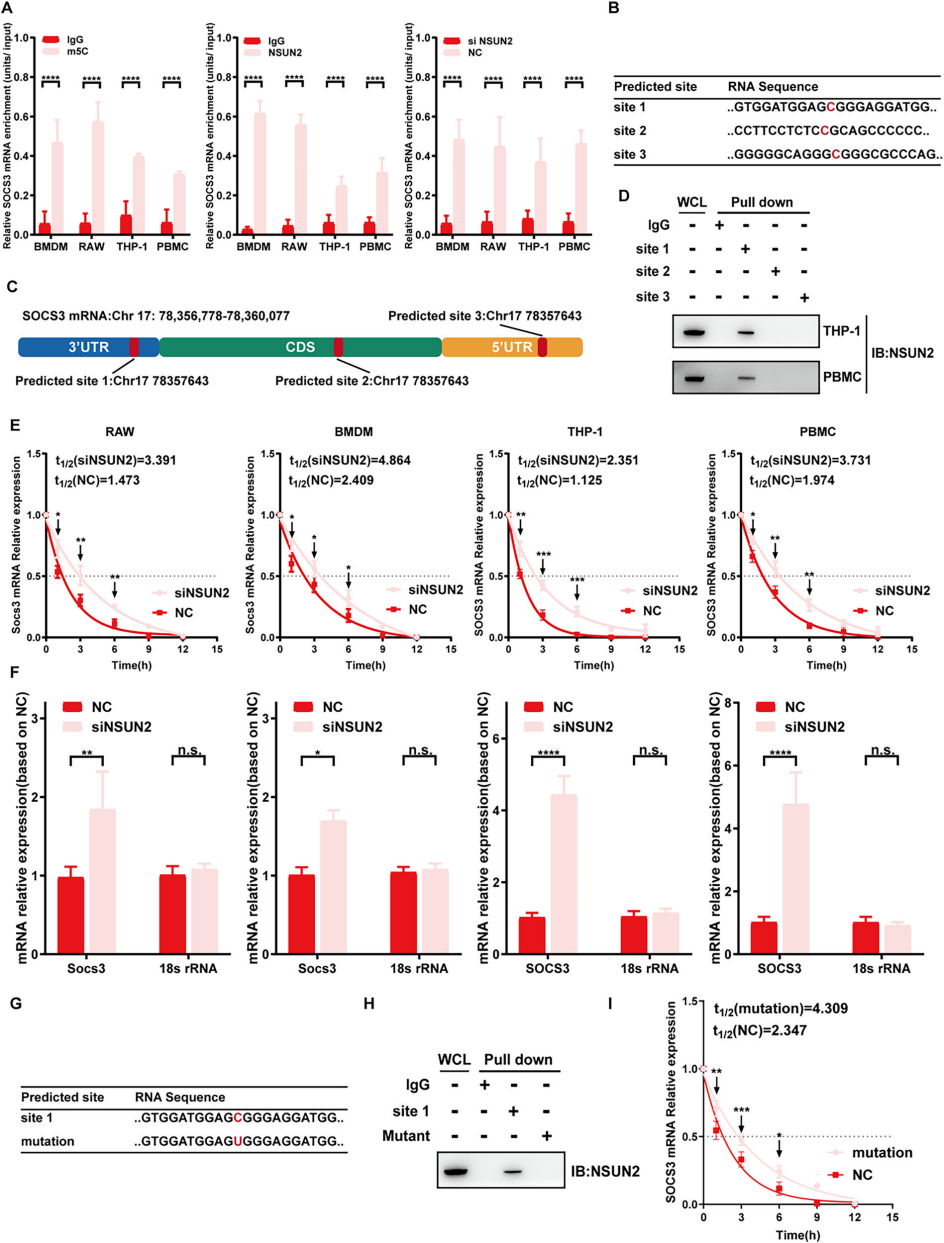
NSUN2 regulates the protein expression of SOCS3 by regulating the stability and nuclear export of SOCS3 mRNA. **A** A series of RIP assays confirmed that the interaction between SOCS3 mRNA and the m5C antibody relies on the presence of NSUN2. **B**, **C** The sequence and location of the predicted m5C sites in SOCS3 mRNA. **D** RNA pull-down assays revealed that site 1 is the m5C site of SOCS3 mRNA. **E** NSUN2 decreases the stability of SOCS3 mRNA. **F** SOCS3 mRNA in the cytoplasm was increased in the siNSUN2 group. **G** The sequence of the mutation SOCS3 mRNA. **H** RNA pull-down assays revealed that NSUN2 cannot bind to the mutation site. **I** The stability of SOCS3 mRNA increased after the mutation.

We wondered how NSUN2 controls the expression of SOCS3. As an m5C RNA methylase, NSUN2 exerts its biological functions by directly binding to targeted mRNAs and methylating determined sites [12]. Thus, we detected whether NSUN2 could directly bind to SOCS3 mRNA via an RNA immunoprecipitation (RIP) assay. As shown in Fig. 7A, RIP detection with the NSUN2 antibody or the m5C antibody significantly pulled down SOCS3 mRNA; however, when NSUN2 was knocked down, the amount of pulled-down SOCS3 mRNA dramatically decreased. These results revealed that NSUN2 could directly bind to SOCS3 mRNA via m5C methylation.

To precisely validate the site of m5C methylation, 3 potential sites of methylation were identified from the m5C prediction website (https://rmvar.renlab.org/) (Fig. 7B and C). Then, primers corresponding to these sites were synthesized, and an RNA pull-down assay was performed for the NSUN2-SOCS3 mRNA complex. The results revealed that both the NSUN2 and m5C proteins could be enriched in the site 1 groups, which indicated that NSUN2 functions by binding to sites 1 (Fig. 7D).

### NSUN2 inhibited the nuclear export of SOCS3 mRNA

Zhang et al. reported that m5C methylation can regulate the stability and nuclear export of targeted mRNAs, which leads to changes in determined mRNA and protein expression [22]. To validate whether NSUN2 mediates SOCS3 expression by affecting the stability or nuclear export of its mRNA, we performed an mRNA stability assay and found that the knockdown of NSUN2 decreased the stability of SOCS3 mRNA (Fig. 7E). Furthermore, we detected the levels of SOCS3 mRNA in the cytoplasm and nucleus and found that the knockdown of NSUN2 led to a significant increase in SOCS3 mRNA in the cytoplasm but a decrease in SOCS3 mRNA in the nucleus (Fig. 7F). To further explore the relationship between NSUN2-mediated m5C methylation and destabilization of SOCS3 mRNA, mutation assay is performed. According to the result, mutation of m5C site 1 prevented the binding of NSUN2 and increased the stability of SOCS3 mRNA eventually (Fig. 7H, I). FISH analysis further confirmed that the knockdown of NSUN2 reduced the accumulation of SOCS3 mRNA in the nucleus (Fig. S5). Taken together, these results suggest that NSUN2-mediated m5C methylation inhibits the nuclear export and stability of SOCS3 mRNA, which leads to the decrease of SOCS3 expression and reduces its inhibition for JAK2/STAT3 pathway eventually.

### NSUN2-mediated SOCS3 mRNA methylation requires the assistance of YBX1 and TET2

**Fig. 8:**
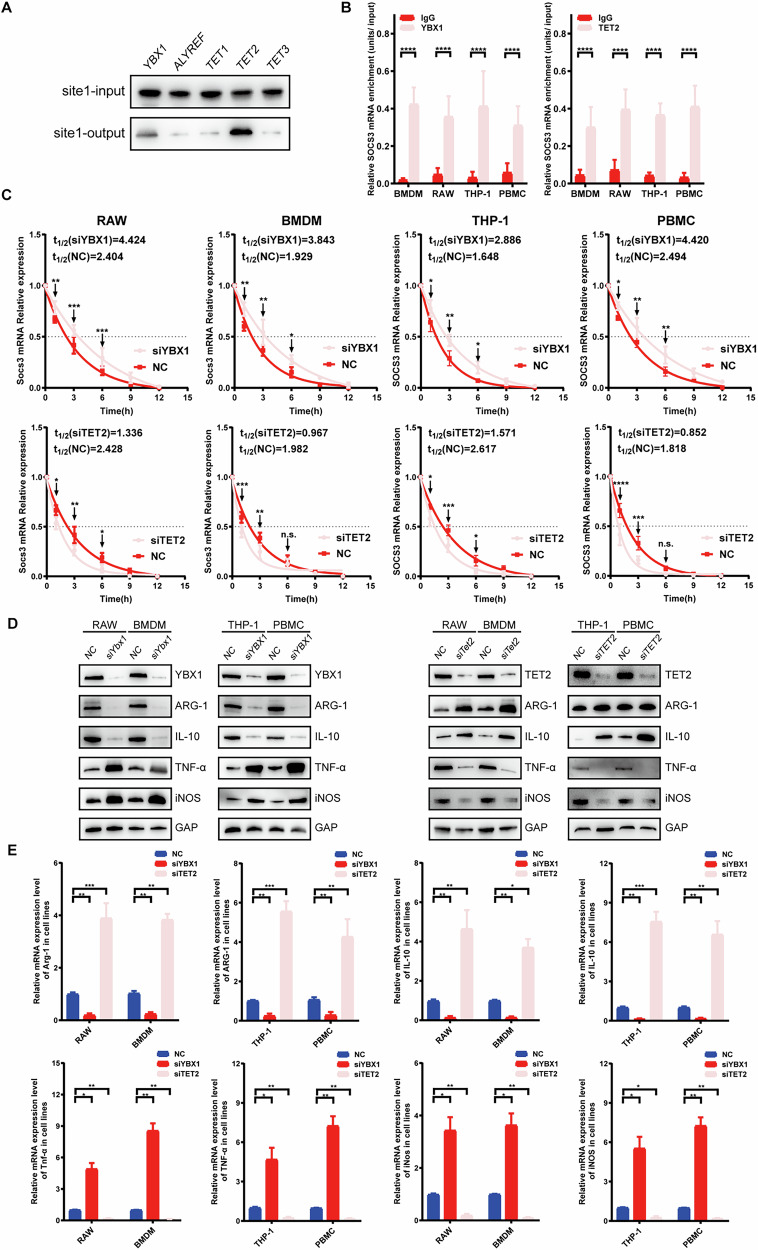
NSUN2-mediated SOCS3 mRNA methylation requires the assistance of the m5C reader YBX1 and the m5C eraser TET2. **A** RNA pull-down assays revealed that the m5C reader YBX1 and the m5C eraser TET2 can also be pulled down by the validated m5C sites 1 and 3. **B** RIP assays revealed that SOCS3 mRNA can be enriched by both YBX1 and TET2 antibodies. **C** Knockdown of YBX1 improved the stability of SOCS3 mRNA, whereas knockdown of TET2 decreased its stability. **D**, **E** Knockdown of YBX1 decreased the mRNA and protein expression of M2-related genes and increased the expression of M1-related genes. In addition, TET2 knockdown resulted in the opposite trend.

The process of NSUN2-mediated m5C methylation usually requires a balance of readers and erasers. Yang et al. reported that NSUN2-mediated RNA m5C methylation requires the assistance of ALYREF (a reader) in the nucleus, facilitating the export of m5C-modified mRNAs [23]. Similarly, Y-box binding protein 1 (YBX1) is also a reader of NSUN2. To determine which types of regulators positively participate in the process of SOCS3 mRNA methylation, RNA oligo pull-down assays were performed on all m5C readers (ALYREF and YBX1) and the m5C eraser (TET1-3) with validated m5C sites of NSUN2 in SOCS3 mRNA. Both YBX1 and TET2 were pulled down at sites 1 (Fig. 8A). Further RIP assays with YBX1 or TET2 antibodies confirmed that SOCS3 mRNA could be pulled down by both antibodies (Fig. 8B).

To define the roles of YBX1 and TET2 in the process of NSUN2-mediated SOCS3 mRNA methylation, either YBX1 or TET2 was knocked down using siRNA technology. The results revealed that siYBX1 resulted in decreased stability of SOCS3 mRNA, whereas siTET2 led to the opposite results (Fig. 8C). Furthermore, a series of RIP assays confirmed that YBX1 and TET2 directly bind to SOCS3 mRNA. To further validate their role in mediating m5C methylation of SOCS3, we performed additional functional experiments. Immunofluorescence analysis demonstrated that knockout of TET2 reduced the global m5C methylation level in macrophages, while subsequent reconstitution of TET2 expression restored it. Meanwhile, RNA stability assays revealed that knockdown of YBX1 up-regulated the stability of SOCS3 mRNA (Fig. S6). Taken together, these results suggest that YBX1 and TET2 participate in the regulation of NSUN2-mediated SOCS3 mRNA methylation.

Additionally, we detected the roles of YBX1 and TET2 in macrophage polarization. As shown in Figs. 8D, E and S5, siYBX1 promoted M1 polarization in macrophages and inhibited M2 polarization, whereas siTET2 had the opposite effect.

## Discussion

TAMs are important components of the tumor microenvironment, and they nurture the course of tumorigenesis and development, although the underlying mechanism is not fully understood [3, 24**]**. In recent decades, the roles of TAMs have been widely explored. M1 and M2 macrophages are two active states of macrophages under various conditions; M2 macrophages have protumor effects, and M1 macrophages have proinflammatory and antitumor effects [5]. A series of studies have shown that M2 macrophages constitute the major percentage of macrophages in solid human cancers [1, 5, 25]. Consistent with these findings, our results also suggest that M2 macrophages constitute the main subtype of TAMs in the bladder TME. However, the states of M1 and M2 are plastic, and they can mutually transform into one another under specific conditions. To clarify this phenomenon, we need to elucidate the underlying mechanism by which macrophages polarize to the M1/M2 phenotype, which could serve as a target for exploring new drugs for cancers in the future.

Epigenetic modifications, including RNA methylation, are critical for the regulation of gene expression, and their roles in the process of cancer development are widely known [12, 26, 27]. However, the role of RNA methylation in the regulation of macrophage polarization has not been sufficiently investigated. m6A is an important form of RNA methylation, and its role in macrophage polarization has been emphasized in recent studies [27–29]. It has been described as a rapid and dynamic “responsive” regulator, which broadly and agilely reshaping the transcriptome landscape of immune-related genes to bidirectionally modulate the M1/M2 equilibrium [11, 27]. On the other hand, another form of RNA methylation, m5C, is generally considered more stable. Its regulation depends more on the expression levels of its writers and readers [30, 31]. However, few studies have revealed the role of m5C in macrophage polarization. Recent studies revealed that RNA modification plays an important role in the regulation of the TME, especially the role of m6A in the polarization of M2 macrophages, whereas studies that focus on m5C in macrophage polarization are insufficient [29, 32]. Additionally, Zhou et al. revealed that m5C in BCa cancer cells promotes the pathogenesis of BCa [33].

Since then, approximately 10 RNA m5C methylases have been identified. To determine which m5C methylase participates in the regulation of macrophage polarization in the bladder TME, we used a qPCR assay to detect their expression in BC tumors surrounding normal tissues. NSUN2 was subsequently identified as the most significant regulator of these known m5C RNA methylases. NSUN2 is the main member of the RNA m5C methylases and catalyzes the methylation of cytosine residues in tRNAs and other RNA molecules, playing a key role in cell differentiation and development [13, 26, 34, 35]. Additionally, further experiments revealed that NSUN2 is related to poor prognosis in BCa patients and M2 polarization in various macrophages. Taken together, these findings indicate that the hypermethylation of m5C in macrophages might trigger M2 polarization in the BCa TME. Although the ability of m5C to promote cancer progression has been well elucidated in cancers, its function in the process of macrophage polarization is still unclear [26, 35]. By knocking down its expression in macrophages, we found that the state of macrophages changed and tended toward M1 polarization, accompanied by elevated expression of M1-related genes. These results suggest that NSUN2 participates in the regulation of M2 polarization.

We subsequently reported that SOCS3 (suppressor of cytokine signaling 3) is the downstream target of NSUN2 and that NSUN2 regulates SOCS3 protein expression via the methylation of SOCS3 mRNA. SOCS3 is a negative regulatory protein that primarily modulates immune responses, inflammation, and metabolic processes by inhibiting the JAK-STAT signaling pathway [18, 36]. It plays a crucial role in maintaining the cellular signaling balance and preventing excessive inflammation and immune responses [18, 37]. Usually, mRNA methylation occurs in 3’ or 5’ untranslated regions (UTRs), and methylation can affect the stability and nuclear export of modified mRNAs [38, 39]. In our study, knockdown of NSUN2 resulted in the upregulation of SOCS3 protein expression as well as its mRNA levels, indicating that NSUN2-mediated m5C modification decreased the stability of SOCS3 mRNA. Next, we detected whether SOCS3 mRNA export was affected after m5C methylation. Consistent with reports that NSUN2-mediated m5C modification can affect the nuclear export of modified mRNAs, the repression of NSUN2 increased the nuclear export of SOCS3 mRNA. We further explored the downstream signaling pathways by which NSUN2 regulates TAM polarization, and the SOCS3/JAK2/STAT3 pathway was inhibited after NSUN2 knockdown. The JAK2/STAT3 pathway is the core pathway involved in TAM polarization in macrophages, and it has been reported that IL-4 regulates M2 polarization mainly by activating the JAK/STAT pathway [40, 41]. Additionally, the upregulation of SOCS3, an upstream inhibitory molecule, is the main cause of the inhibition of the JAK2/STAT3 pathway.

**Fig. 9:**
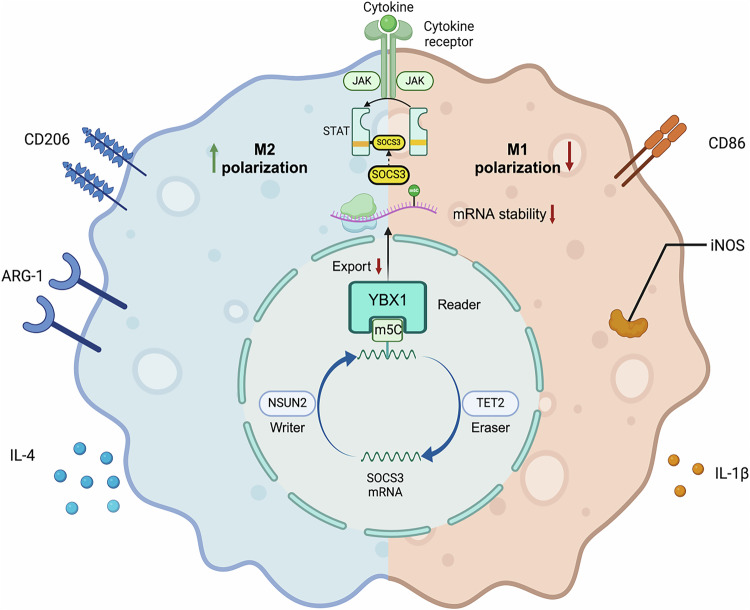
Schematic diagram. NSUN2 promotes M2 polarization of TAMs by methylating SOCS3 mRNA, thereby activating the JAK2/STAT3 signaling pathway. Additionally, m5C methylation in BCa regulates the M1/M2 polarization of TAMs through the NSUN2-m5C-YBX1/TET2 axis.

m5C modification not only mediates the binding of mRNAs through m5C writers but also exerts regulatory effects through the combination of m5C readers and erasers, which can recognize and bind to m5C sites and produce biological effects [13, 42]. In this study, we found that the expression of YBX1, ALYREF (m5C readers), and TET2 (m5C eraser) changed significantly in BCa TAMs compared with those in normal tissues, which was also related to the expression of NSUN2. YBX1, as a reader, recognizes and binds to specific RNA modifications, influencing RNA stability, translation, and processing,g and thereby regulating gene expression [42]. On the other hand, TET2, as an eraser, removes certain RNA modifications by oxidizing methylated cytosine residues, thereby facilitating dynamic changes in RNA function and allowing the fine-tuning of cellular processes such as differentiation and stress responses [43, 44]. Together, these two proteins coordinate the regulation of RNA modifications, ensuring proper cellular function and adaptation. Further results of RNA oligo pull-down verified that YBX1 and TET2 could bind to the predicted m5C sites in SOCS3 mRNA that also bind to NSUN2, and that the combination of YBX1 and TET2 with m5C sites relies on the expression of NSUN2. Furthermore, YBX1 knockdown increased SOCS3 mRNA stability, whereas TET2 knockdown decreased SOCS3 mRNA stability, which ultimately contributed to changes in the SOCS3/JAK2/STAT3 pathway. These results suggest that SOCS3 mRNA can be m5C modified by NSUN2, which destabilizes SOCS3 mRNA with the assistance of YBX1, combined with its m5C sites, promoting the activation of the JAK2/STAT3 pathway. On the other hand, TET2 can reverse the effect of NSUN2 by regulating the demethylation of mRNA, converting the abnormal activation of the downstream pathway into inhibition. Through the above mechanism, the activation/inhibition of the JAK2/STAT3 pathway regulates the polarization of BCa TAMs, which ultimately mediates the progression of BCa (Fig. 9).

Therefore, these findings suggest that the NSUN2-m5C-YBX1/TET2 axis in BCa TAMs regulates the polarization of macrophages in the TME of BCa and ultimately affects the prognosis of BCa patients. By intervening in and monitoring this axis artificially, we might predict BCa patient prognosis individually, predict the effectiveness of immunotherapy or interfere with the progression of BCa. Unfortunately, there are no available medical antibodies or inhibitors for NSUN2 on the market. However, Jiang et al. reported small molecule inhibitors that could decrease the methyltransferase activity of NSUN2, which could provide new therapeutic options in the future [45]. Similarly, to the best of our knowledge, research has yet to identify an agonist of TET2, which is expressed at low levels or inactivated in various cancers. On the other hand, YBX1 has been well studied as a common cancer-associated antigen [46–48**]**. YBX1 can induce a T-cell response and immune evasion by regulating PD-L1, and studies have shown that YBX1 can be inhibited by everolimus and the YBX1 phosphorylation inhibitor TAS0612 [49, 50]. Therefore, targeting YBX1 in this axis might be a new strategy for treating advanced BCa patients.

In conclusion, we found that M2 polarization and m5C modification were significantly increased in BCa TAMs. Further mechanistic research revealed that the NSUN2-m5C-YBX1/TET2 axis could regulate SOCS3 mRNA and further activate the JAK2/STAT3 pathway, leading to M2 polarization of TAMs (Fig. 9). These findings suggest that the NSUN2-m5C-YBX1/TET2 axis could be an ideal target for immunotherapy in BCa patients.

## Supplementary information

Supplementary materials

WB raw image--supplementary materials

## Data Availability

The datasets supporting the conclusions of this article are available from the corresponding author on reasonable request. The authenticity of this article has been validated by uploading the key raw data onto the Research Data Deposit platform (www.researchdata.org.cn).

## References

[1] Boutilier AJ, Elsawa SF. Macrophage polarization states in the tumor microenvironment. Int J Mol Sci. 2021;22:6995. 10.3390/ijms22136995 PMC8268869 34209703

[2] Sica A, Mantovani A. Macrophage plasticity and polarization: in vivo veritas. J Clin Investig. 2012;122:787–95. 22378047 10.1172/JCI59643 PMC3287223

[3] Tardito S, Martinelli G, Soldano S, Paolino S, Pacini G, Patane M, et al. Macrophage M1/M2 polarization and rheumatoid arthritis: A systematic review. Autoimmun Rev. 2019;18:102397. 10.1016/j.autrev.2019.102397 31520798

[4] Van den Bossche J, Baardman J, Otto NatasjaA, van der Velden S, Neele AnnetteE, van den Berg SusanM, et al. Mitochondrial dysfunction prevents repolarization of inflammatory macrophages. Cell Rep. 2016;17:684–96. 27732846 10.1016/j.celrep.2016.09.008

[5] Pittet MJ, Michielin O, Migliorini D. Clinical relevance of tumour-associated macrophages. Nature Rev Clin Oncol. 2022;19:402–21. 35354979 10.1038/s41571-022-00620-6

[6] Wang Y, Yan K, Wang J, Lin J, Bi J. M2 Macrophage co-expression factors correlate with immune phenotype and predict prognosis of bladder cancer. Front Oncol. 2021;11:609334. 10.3389/fonc.2021.609334 PMC8019942 33828973

[7] Han S, Wang W, Wang S, Yang T, Zhang G, Wang D, et al. Tumor microenvironment remodeling and tumor therapy based on M2-like tumor associated macrophage-targeting nano-complexes. Theranostics. 2021;11:2892–916. 33456579 10.7150/thno.50928 PMC7806477

[8] Liu Y, Liu Z, Tang H, Shen Y, Gong Z, Xie N, et al. The N(6)-methyladenosine (m(6)A)-forming enzyme METTL3 facilitates M1 macrophage polarization through the methylation of STAT1 mRNA. Am J Physiol Cell Physiol. 2019;317:C762–c75. 31365297 10.1152/ajpcell.00212.2019

[9] Li X, Ma S, Deng Y, Yi P, Yu J. Targeting the RNA m(6)A modification for cancer immunotherapy. Mol Cancer. 2022;21:76. 35296338 10.1186/s12943-022-01558-0 PMC8924732

[10] Du J, Liao W, Liu W, Deb DK, He L, Hsu PJ, et al. N(6)-Adenosine Methylation of Socs1 mRNA is required to sustain the negative feedback control of macrophage activation. Dev Cell. 2020;55:737–53.e7. 33220174 10.1016/j.devcel.2020.10.023 PMC7755741

[11] Ma S, Sun B, Duan S, Han J, Barr T, Zhang J, et al. YTHDF2 orchestrates tumor-associated macrophage reprogramming and controls antitumor immunity through CD8(+) T cells. Nat Immunol. 2023;24:255–66. 36658237 10.1038/s41590-022-01398-6 PMC10150872

[12] Chen B, Xi Y, Zhao J, Hong Y, Tian S, Zhai X, et al. m5C regulator-mediated modification patterns and tumor microenvironment infiltration characterization in colorectal cancer: One step closer to precision medicine. Front Immunol. 2022;13:1049435. 10.3389/fimmu.2022.1049435 PMC9751490 36532062

[13] Wang N, Chen R-X, Deng M-H, Wei W-S, Zhou Z-H, Ning K, et al. m5C-dependent cross-regulation between nuclear reader ALYREF and writer NSUN2 promotes urothelial bladder cancer malignancy through facilitating RABL6/TK1 mRNAs splicing and stabilization. Cell Death Dis. 2023;14:139. 10.1038/s41419-023-05661-y PMC9938871 36806253

[14] Fang L, Huang H, Lv J, Chen Z, Lu C, Jiang T, et al. m5C-methylated lncRNA NR_033928 promotes gastric cancer proliferation by stabilizing GLS mRNA to promote glutamine metabolism reprogramming. Cell Death Dis. 2023;14:520. 37582794 10.1038/s41419-023-06049-8 PMC10427642

[15] Song H, Zhang J, Liu B, Xu J, Cai B, Yang H, et al. Biological roles of RNA m(5)C modification and its implications in Cancer immunotherapy. Biomarker Res. 2022;10:15. 10.1186/s40364-022-00362-8 PMC8973801 35365216

[16] Liu K, Zhao E, Ilyas G, Lalazar G, Lin Y, Haseeb M, et al. Impaired macrophage autophagy increases the immune response in obese mice by promoting proinflammatory macrophage polarization. Autophagy. 2015;11:271–84. 25650776 10.1080/15548627.2015.1009787 PMC4502775

[17] Li Y, Mateu E, Díaz I. Impact of Cryopreservation on viability, phenotype, and functionality of porcine PBMC. Front Immunol. 2021;12:765667. 10.3389/fimmu.2021.765667 PMC8666977 34912338

[18] Carow B, Rottenberg ME. SOCS3, a major regulator of infection and inflammation. Front Immunol. 2014;5:58. 10.3389/fimmu.2014.00058 PMC3928676 24600449

[19] Runtsch MC, Angiari S, Hooftman A, Wadhwa R, Zhang Y, Zheng Y, et al. Itaconate and itaconate derivatives target JAK1 to suppress alternative activation of macrophages. Cell Metab. 2022;34:487–501.e8. 35235776 10.1016/j.cmet.2022.02.002

[20] Cui Y, Chen C, Tang Z, Yuan W, Yue K, Cui P, et al. TREM2 deficiency aggravates renal injury by promoting macrophage apoptosis and polarization via the JAK-STAT pathway in mice. Cell Death Dis. 2024;15:401. 38849370 10.1038/s41419-024-06756-w PMC11161629

[21] Zhao Y, Peng F, He J, Qu Y, Ni H, Wu L, et al. SOCS1 Peptidomimetic alleviates glomerular inflammation in MsPGN by inhibiting macrophage M1 polarization. Inflammation. 2023;46:2402–14. 37581761 10.1007/s10753-023-01886-3

[22] Zhang X, Liu Z, Yi J, Tang H, Xing J, Yu M, et al. The tRNA methyltransferase NSun2 stabilizes p16INK4 mRNA by methylating the 3′-untranslated region of p16. Nat Commun. 2012;3:712. 10.1038/ncomms1692 PMC3509206 22395603

[23] Yang X, Yang Y, Sun B-F, Chen Y-S, Xu J-W, Lai W-Y, et al. 5-methylcytosine promotes mRNA export — NSUN2 as the methyltransferase and ALYREF as an m5C reader. Cell Res. 2017;27:606–25. 28418038 10.1038/cr.2017.55 PMC5594206

[24] Xu Y, Zeng H, Jin K, Liu Z, Zhu Y, Xu L, et al. Immunosuppressive tumor-associated macrophages expressing interleukin-10 conferred poor prognosis and therapeutic vulnerability in patients with muscle-invasive bladder cancer. J ImmunoTher Cancer. 2022;10:e003416. 10.1136/jitc-2021-003416 PMC8961180 35338085

[25] Liu KX, Joshi S. “Re-educating” Tumor associated macrophages as a novel immunotherapy strategy for neuroblastoma. Front Immunol. 2020;11:1947. 10.3389/fimmu.2020.01947 PMC7493646 32983125

[26] Chen T, Xu Z-G, Luo J, Manne RK, Wang Z, Hsu C-C, et al. NSUN2 is a glucose sensor suppressing cGAS/STING to maintain tumorigenesis and immunotherapy resistance. Cell Metab. 2023;35:1782–98.e8. 37586363 10.1016/j.cmet.2023.07.009 PMC10726430

[27] Yin H, Zhang X, Yang P, Zhang X, Peng Y, Li D, et al. RNA m6A methylation orchestrates cancer growth and metastasis via macrophage reprogramming. Nat Commun. 2021;12:1394. 10.1038/s41467-021-21514-8 PMC7925544 33654093

[28] Zhuang T, Chen MH, Wu RX, Wang J, Hu XD, Meng T, et al. ALKBH5-mediated m6A modification of IL-11 drives macrophage-to-myofibroblast transition and pathological cardiac fibrosis in mice. Nat Commun. 2024;15:1995. 38443404 10.1038/s41467-024-46357-x PMC10914760

[29] Wang X, Ji Y, Feng P, Liu R, Li G, Zheng J, et al. The m6A Reader IGF2BP2 Regulates Macrophage Phenotypic Activation and Inflammatory Diseases by Stabilizing TSC1 and PPARγ. Advanced Sci. 2021;8:2100209. 10.1002/advs.202100209 PMC8261491 34258163

[30] Wang Z, Mierxiati A, Zhu W, Li T, Xu H, Wan F, et al. FOXA1-dependent NSUN2 facilitates the advancement of prostate cancer by preserving TRIM28 mRNA stability in a m5C-dependent manner. NPJ Precis Oncol. 2025;9:127. 40319192 10.1038/s41698-025-00904-x PMC12049421

[31] Qi Q, Zhong R, Huang Y, Tang Y, Zhang XW, Liu C, et al. The RNA M5C methyltransferase NSUN2 promotes progression of hepatocellular carcinoma by enhancing PKM2-mediated glycolysis. Cell Death Dis. 2025;16:82. 39924557 10.1038/s41419-025-07414-5 PMC11808121

[32] Han X, Liu L, Huang S, Xiao W, Gao Y, Zhou W, et al. RNA m(6)A methylation modulates airway inflammation in allergic asthma via PTX3-dependent macrophage homeostasis. Nat Commun. 2023;14:7328. 37957139 10.1038/s41467-023-43219-w PMC10643624

[33] Chen X, Li A, Sun BF, Yang Y, Han YN, Yuan X, et al. 5-methylcytosine promotes pathogenesis of bladder cancer through stabilizing mRNAs. Nat Cell Biol. 2019;21:978–90. 31358969 10.1038/s41556-019-0361-y

[34] Wang H, Feng J, Zeng C, Liu J, Fu Z, Wang D, et al. NSUN2-mediated m^5^C methylation of IRF3 mRNA negatively regulates type I interferon responses during various viral infections. Emerg Microbes Infect. 2023;12:2178238. 10.1080/22221751.2023.2178238 PMC9946332 36748584

[35] Zhu W, Wan F, Xu W, Liu Z, Wang J, Zhang H, et al. Positive epigenetic regulation loop between AR and NSUN2 promotes prostate cancer progression. Clin Transl Med. 2022;12:e1028. 10.1002/ctm2.1028 PMC9516604 36169095

[36] Rottenberg ME, Carow B. SOCS3 and STAT3, major controllers of the outcome of infection with Mycobacterium tuberculosis. Seminars Immunol. 2014;26:518–32. 10.1016/j.smim.2014.10.004 25458989

[37] Haider MJA, Albaqsumi Z, Al-Mulla F, Ahmad R, Al-Rashed F. SOCS3 Regulates Dectin-2-induced inflammation in PBMCs of diabetic patients. Cells. 2022;11:2670. 10.3390/cells11172670 PMC9454960 36078084

[38] Lesbirel S, Wilson SA. The m6A‑methylase complex and mRNA export. Biochimica et Biophys Acta (BBA) - Gene Regul Mech. 2019;1862:319–28. 10.1016/j.bbagrm.2018.09.008 PMC6414750 30290229

[39] Lee M, Kim B, Kim VN. Emerging roles of RNA modification: m6A and U-Tail. Cell. 2014;158:980–7. 25171402 10.1016/j.cell.2014.08.005

[40] He Y, Gao Y, Zhang Q, Zhou G, Cao F, Yao S. IL-4 Switches Microglia/macrophage M1/M2 polarization and alleviates neurological damage by modulating the JAK1/STAT6 pathway following ICH. Neuroscience. 2020;437:161–71. 32224230 10.1016/j.neuroscience.2020.03.008

[41] Xu Y, Liu J, Wang J, Wang J, Lan P, Wang T. USP25 stabilizes STAT6 to promote IL-4-induced macrophage M2 polarization and fibrosis. Int J Biol Sci. 2025;21:475–89. 39781451 10.7150/ijbs.99345 PMC11705635

[42] Liu L, Chen Y, Zhang T, Cui G, Wang W, Zhang G, et al. YBX1 Promotes esophageal squamous cell carcinoma progression via m5C-dependent SMOX mRNA stabilization. Adv Sci. 2024;11:e2302379. 10.1002/advs.202302379 PMC11132058 38566431

[43] Zou Z, Dou X, Li Y, Zhang Z, Wang J, Gao B, et al. RNA m(5)C oxidation by TET2 regulates chromatin state and leukaemogenesis. Nature. 2024;634:986–94. 39358506 10.1038/s41586-024-07969-x PMC11499264

[44] Moran-Crusio K, Reavie L, Shih A, Abdel-Wahab O, Ndiaye-Lobry D, Lobry C, et al. Tet2 loss leads to increased hematopoietic stem cell self-renewal and myeloid transformation. Cancer Cell. 2011;20:11–24. 21723200 10.1016/j.ccr.2011.06.001 PMC3194039

[45] Chen B, Deng Y, Hong Y, Fan L, Zhai X, Hu H, et al. Metabolic recoding of NSUN2-Mediated m(5)C modification promotes the progression of colorectal cancer via the NSUN2/YBX1/m(5)C-ENO1 positive feedback loop. Adv Sci. 2024;11:e2309840. 10.1002/advs.202309840 PMC11267267 38769664

[46] Goodarzi H, Liu X, Nguyen HC, Zhang S, Fish L, Tavazoie SF. Endogenous tRNA-derived fragments suppress breast cancer progression via YBX1displacement. Cell. 2015;161:790–802. 25957686 10.1016/j.cell.2015.02.053 PMC4457382

[47] Liu B, Shen H, He J, Jin B, Tian Y, Li W, et al. Cytoskeleton remodeling mediated by circRNA-YBX1 phase separation suppresses the metastasis of liver cancer. Proc Natl Acad Sci USA. 2023;120:e2220296120. 37459535 10.1073/pnas.2220296120 PMC10372620

[48] Meng H, Miao H, Zhang Y, Chen T, Yuan L, Wan Y, et al. YBX1 promotes homologous recombination and resistance to platinum-induced stress in ovarian cancer by recognizing m5C modification. Cancer Lett. 2024;597:217064. 38880223 10.1016/j.canlet.2024.217064

[49] Xi Q, Yang G, He X, Zhuang H, Li L, Lin B, et al. M(6)A-mediated upregulation of lncRNA TUG1 in liver cancer cells regulates the antitumor response of CD8( + ) T cells and phagocytosis of macrophages. Adv Sci. 2024;11:e2400695. 10.1002/advs.202400695 PMC11425850 38981064

[50] Shibata T, Watari K, Kawahara A, Sudo T, Hattori S, Murakami Y, et al. Targeting phosphorylation of Y-Box-binding protein YBX1 by TAS0612 and Everolimus in overcoming antiestrogen resistance. Mol Cancer Ther. 2020;19:882–94. 31879363 10.1158/1535-7163.MCT-19-0690

