# Supplementary material for: NSUN2-mediated m5C modification of SOCS3 mRNA modulates macrophage polarization in bladder cancer
Source: Cell Death Dis. 2025 Dec 7;17(1):75. doi: 10.1038/s41419-025-08306-4 (PMC12827474; doi:10.1038/s41419-025-08306-4)
Supplement: Supplementary file 1 — Supplementary materials [file 41419_2025_8306_MOESM1_ESM.pdf]

Antibody	Company	Catalogue number	Dilution	RRID
NSUN2	CST	E9Y5E	1:1000	
GAPDH	CST	D16H11	1:5000	
SOCS3	CST	D6E1T	1:1000	
β-Actin	CST	13E5	1:5000	
Arginase-1	CST	D4E3M	1:1000	
IL-10	Abcam	ab310329	1:1000	
IL-10	Abcam	ab133575	1:1000	
TNF-α	CST	3707	1:1000	
iNOS	CST	D6B6S	1:1000	
JAK2	CST	D2E12	1:1000	
STAT3	CST	124H6	1:1000	
YBX1	Thermo Fisher	9J7M4	1:1000	
ALYREF	Abcam	ab202894	1:1000	
TET1	Abcam	ab191698	1:1000	
TET2	Abcam	ab213369	1:1000	
TET3	Abcam	ab231785	1:1000	

Table S1 Details of used antibodies

Gene name	Forward sequence (5' -> 3')	Reverse sequence (5' -> 3')
NSUN2	GGGTTGCTATCACTCTCTATGC	TTTCTTGTCTGTTGCCGTAGTT
Nsun2	CTCTGTTCAGCTATTGGACGC	TGGCACTCCCAAACATAATTTGA
GAPDH	GGAGCGAGATCCCTCCAAAAT	GGCTGTTGTCATACTTCTCATGG
Gapdh	AGGTCGGTGTGAACGGATTTG	GGGGTCGTTGATGGCAACA
ARG-1	GTGGAAACTTGCATGGACAA	AATCCTGGCACATCGGGAATC
Arg-1	CTCCAAGCCAAAGTCCTTAGAG	GGAGCTGTCATTAGGGACATCA
IL-10	GACTTTAAGGGTTACCTGGGTTG	TCACATGCGCCTTGATGTCTG
Il-10	CTTACTGACTGGCATGAGGATCA	GCAGCTCTAGGAGCATGTGG
TNF-α	CCTCTCTCTAATCAGCCCTCTG	GAGGACCTGGGAGTAGATGAG
Tnf-α	CAGGCGGTGCCTATGTCTC	CGATCACCCCGAAGTTCAGTAG
iNOS	TTCAGTATCACAACCTCAGCAAG	TGGACCTGCAAGTTAAAATCCC
iNos	GTTCTCAGCCCAACAATACAAGA	GTGGACGGGTCGATGTCAC
GAPDH	GGAGCGAGATCCCTCCAAAAT	GGCTGTTGTCATACTTCTCATGG
Gapdh	AATGGATTTGGACGCATTGGT	TTTGCACTGGTACGTGTTGAT

Table S2 Details of used primers.

Gene name	Sense (5' -> 3')	Anti-sense (5' -> 3')
NSUN2	GCGAUGCCUUAGGAUAUUATT	UAAUAUCCUAAGGCAUCGCTT
Nsun2	GCCUGGCAUACAAACUUAATT	UUAAGUUUGUAUGCCAGGCTT
SOCS3	AACAAGUUCCGUUGGAAAGUU	CUUUCCAACGGAACUUGUUUG
Socs3	UCUUGUAAUGUUUAGUCACUA	GUGACUAAACAUUACAAGAAG
YBX1	UUUUACUGUUCCCAAAACCUU	GGUUUUGGGAACAGUAAAAUG
Ybx1	UUACAUUGAACCAUUUGACUG	GUCAAAUGGUUCAAUGUAAGG
TET2	AUAAUAACUUUUGAAAGAGUG	CUCUUUCAAAAGUUAUUAUGG
Tet2	AUUCAUAAGGCAGUUUGACGU	GUCAAACUGCCUUAUGAAUAU

Table S3 effective sequences of used siRNA.

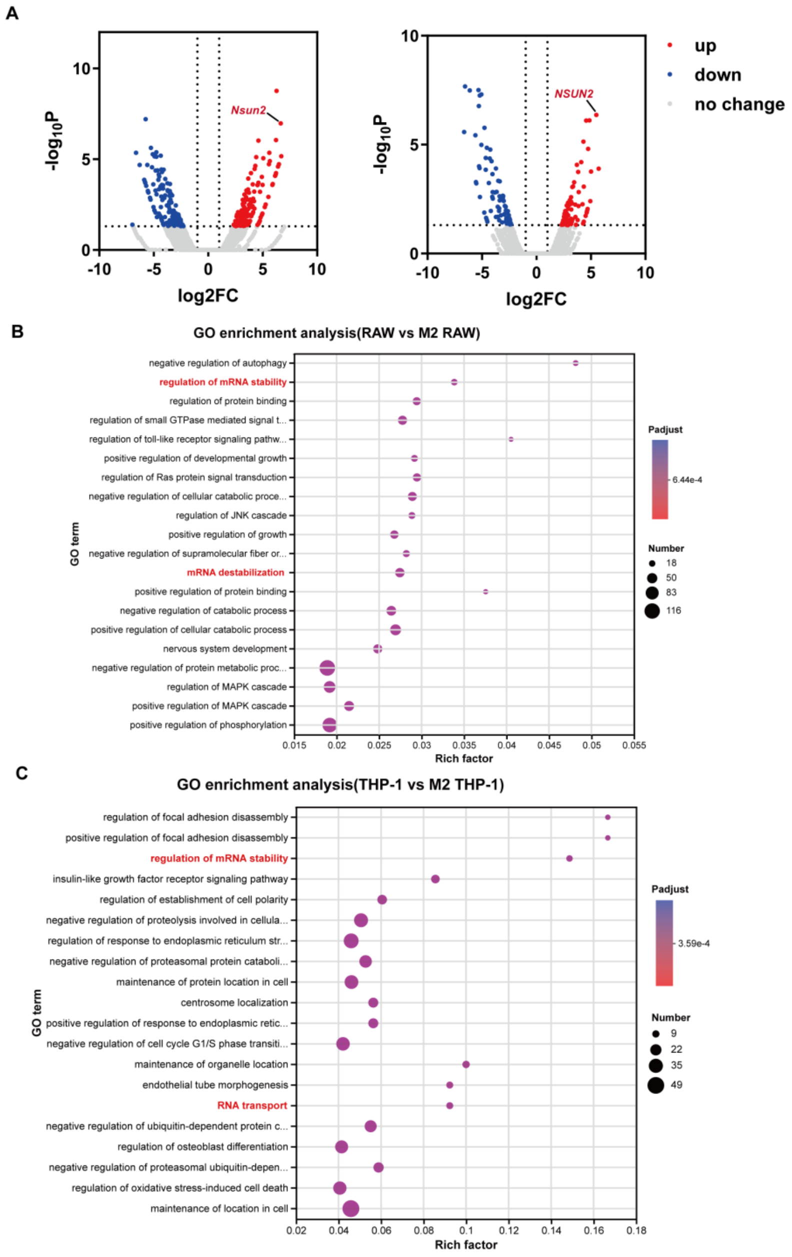

Figure S1 NSUN2 was commonly upregulated in M2 macrophages compared with M0 macrophages (A) and NSUN2-related pathways were enriched in GO analysis (B-C).

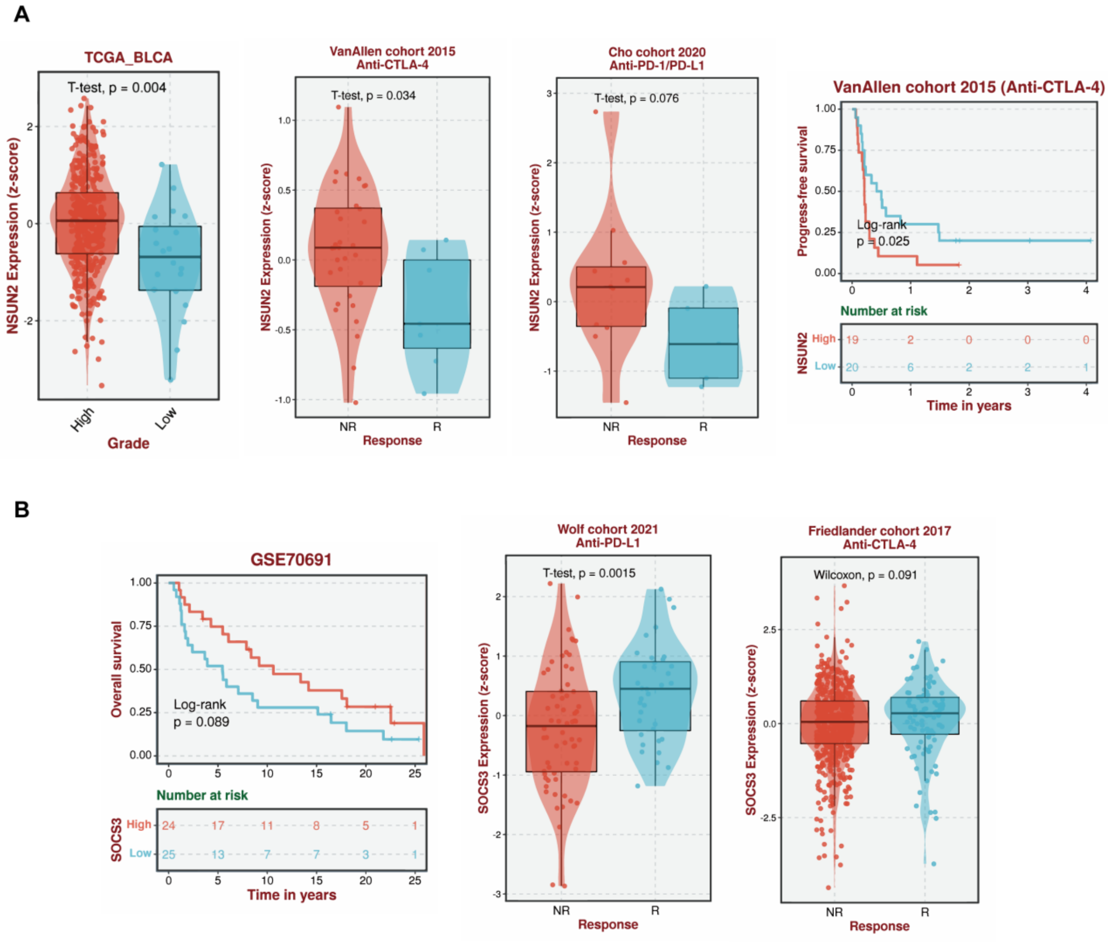

Figure S2 Relevant data from public databases that reveal the relationship between NSUN2/SOCS3 expression and clinical data in Bladder cancer. High NSUN2 expression is associated with a poor response and prognosis to immunotherapy. (A) And high SOCS3 expression is associated with a better response and prognosis to immunotherapy. (B).

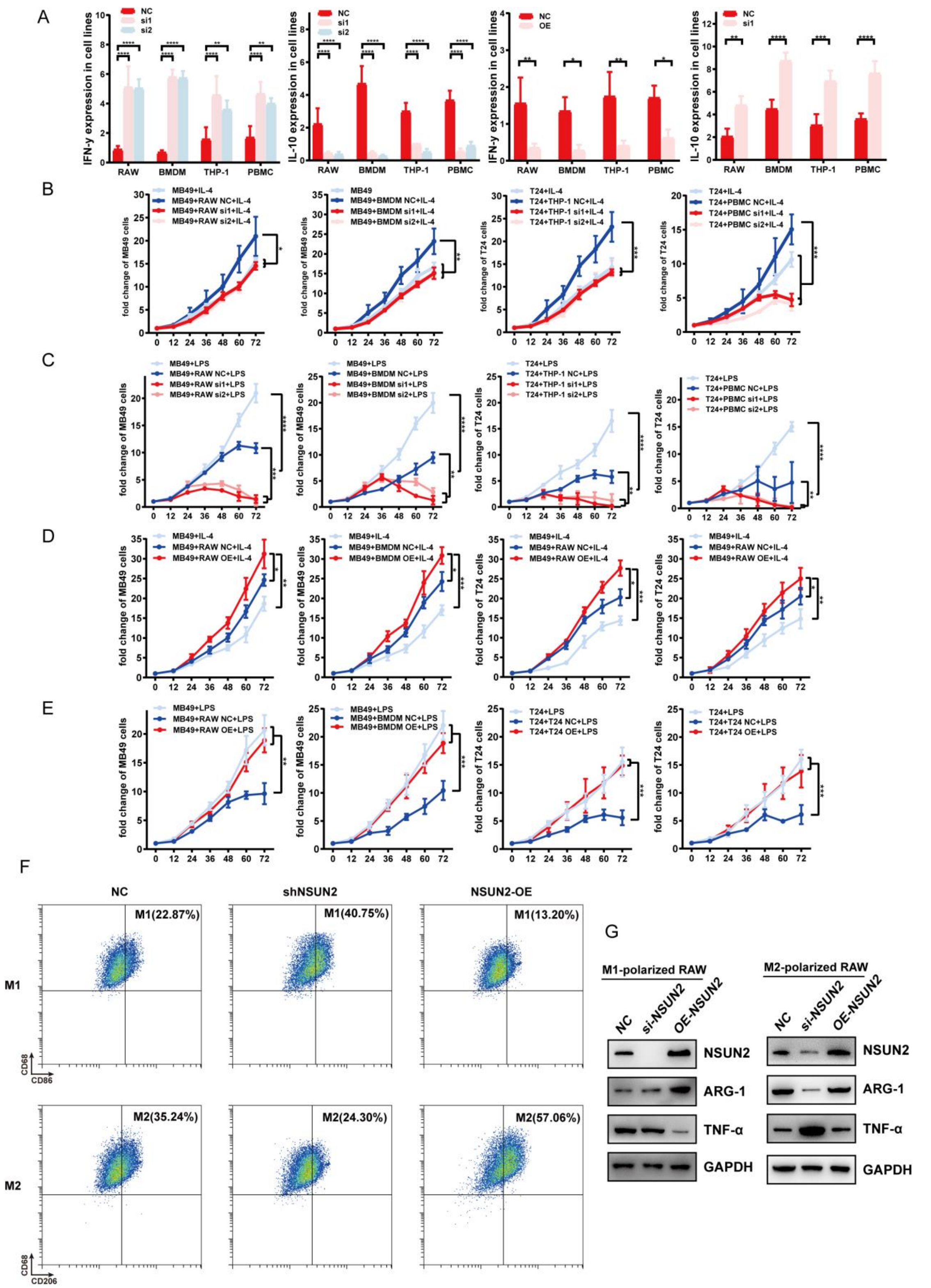

Figure S3 Knockdown of NSUN2 reduced the secretion of IL-10 and promoted the secretion of TNF-α.(A) The knock-down of NSUN2 inhibited the pro-tumor effect of M2 macrophages while promoted the anti-tumor effect of M1 macrophages. (B-C) On the other hand, the over-expression of NSUN2 showed opposite effect.(D-E) The flow cytometry of THP-1 showed that expression of NSUN2 affected the

M1/M2 proprotion of THP-1 after induction of IL-4/LPS.(F)To exclude off-target effects, over-expressing NSUN2 and silencing NSUN2 in M1-polarized /M2-polarized macrophages were performed.(G)

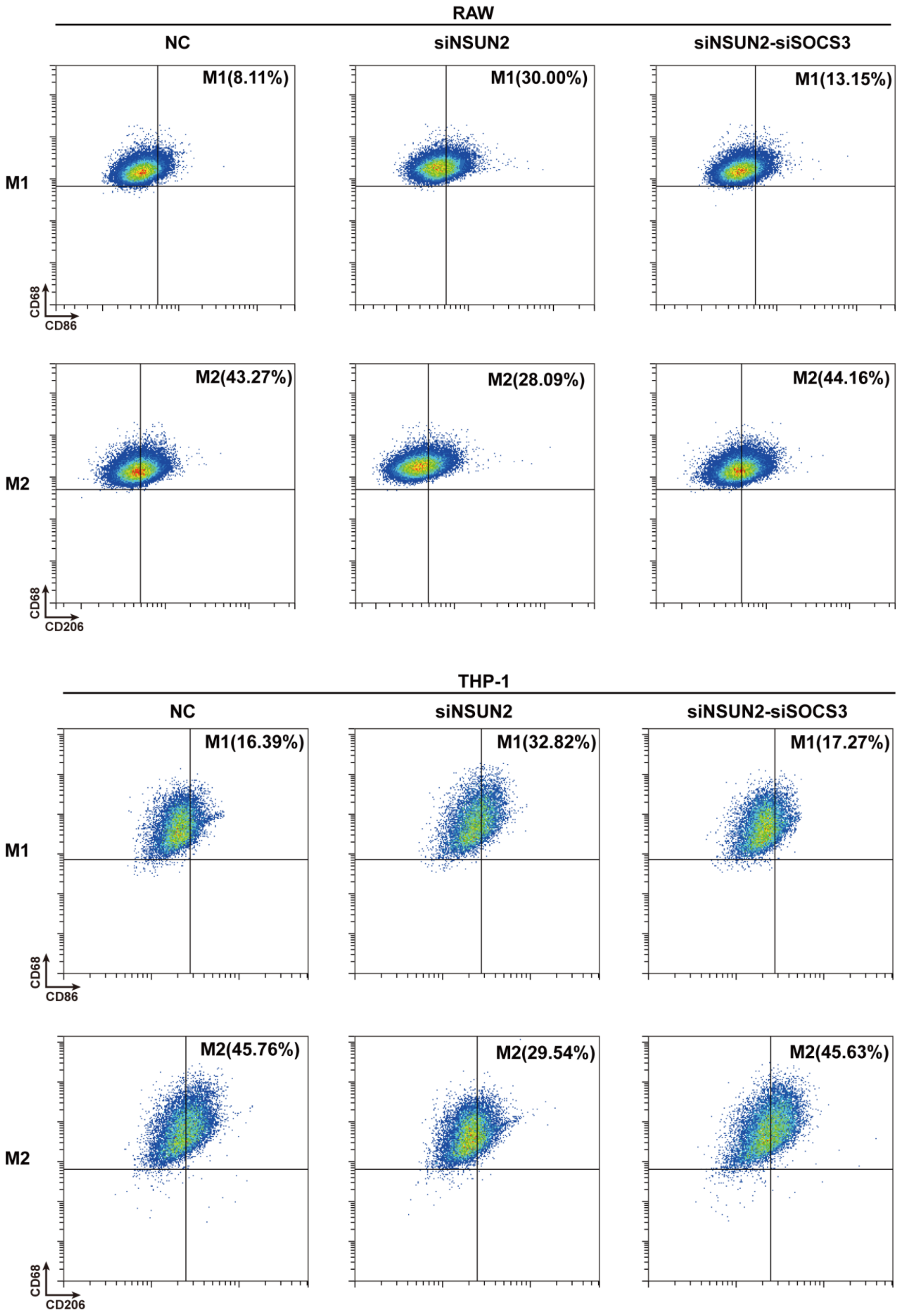

Figure S4 The flow cytometry of THP-1 and RAW improved that knockdown of SOCS3 remodeled the polarization and ability of macrophages caused by NSUN2 inhibition.

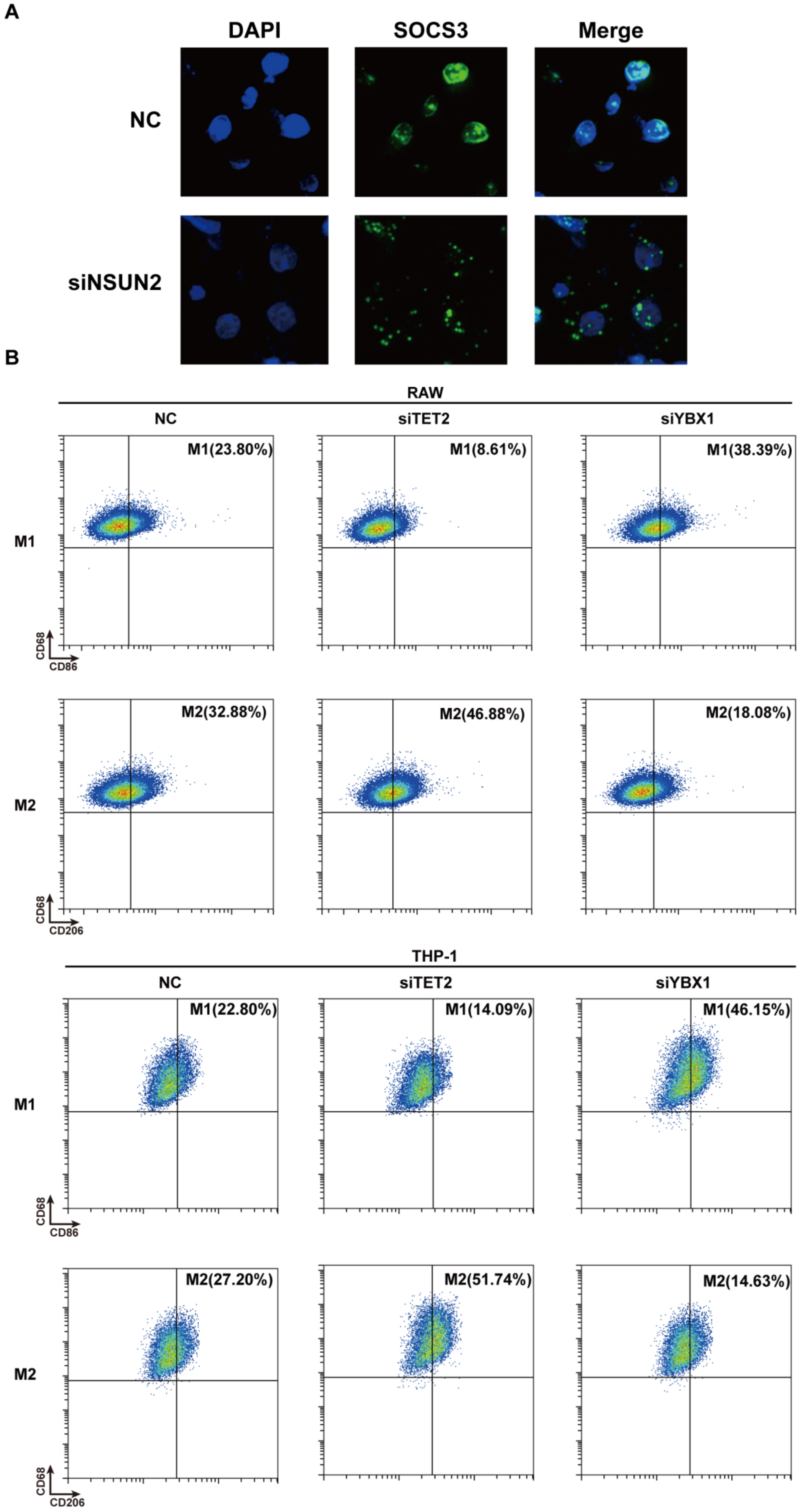
A
DAPI
SOCS3
Merge
NC
siNSUN2
B
RAW
NC
siTET2
siYBX1
M1(23.80%)
M1(8.61%)
M1(38.39%)
M1
CD68
CD86
M2(32.88%)
M2(46.88%)
M2(18.08%)
M2
CD68
CD206
THP-1
NC
siTET2
siYBX1
M1(22.80%)
M1(14.09%)
M1(46.15%)
M1
CD68
CD86
M2(27.20%)
M2(51.74%)
M2(14.63%)
M2
CD68
CD206

Figure S5 The FISH analysis further confirmed that the knockdown of NSUN2 reduced the accumulation of SOCS3 mRNA in the nucleus.(A) The flow cytometry of THP-1 and RAW verified that knockdown of YBX1 promoted M1 polarization in macrophages and inhibited M2 polarization, whereas knockdown of TET2 had the opposite effect.

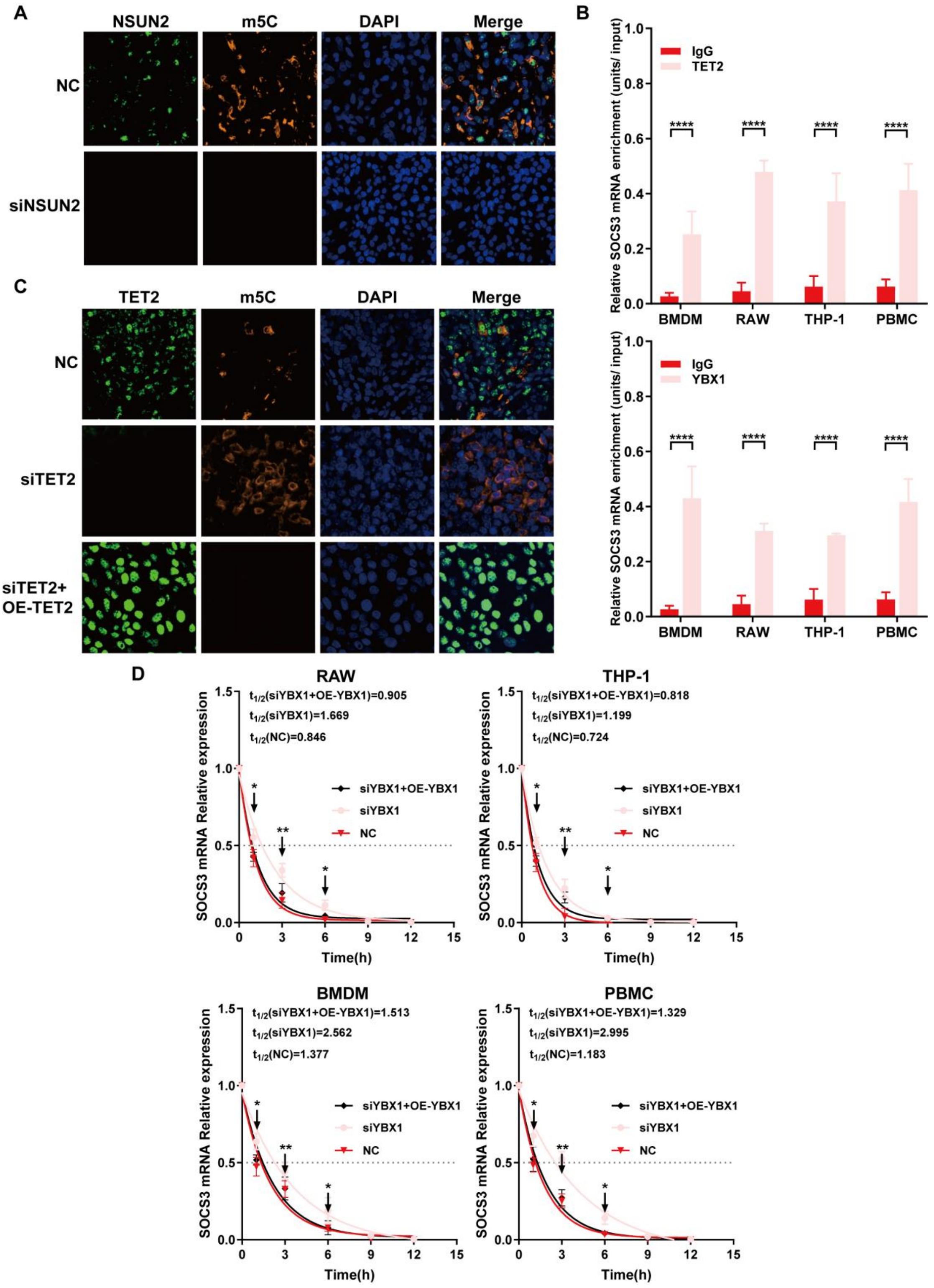

Figure S6 mIF showed that the NSUN2 expression is associated with the level of m5C methylation in macrophages. (A). RIP assays confirmed that the interaction between SOCS3 mRNA and YBX1/TET2 (B). TET2-knockout reduced m5C methylation levels in macrophages, and this reduction was rescued by the subsequent reconstitution of TET2 expression (C). YBX1-knockout increases the stability of SOCS3 mRNA, which was rescued by the subsequent over-expression of YBX1 (D).
