# Supplementary material for: NSUN2-mediated m5C modification of SOCS3 mRNA modulates macrophage polarization in bladder cancer
Source: Cell Death Dis. 2025 Dec 7;17(1):75. doi: 10.1038/s41419-025-08306-4 (PMC12827474; doi:10.1038/s41419-025-08306-4)
Supplement: Supplementary file 2 — WB raw image--supplementary materials [file 41419_2025_8306_MOESM2_ESM.docx]

Figure2---A (RAW+BMDM)


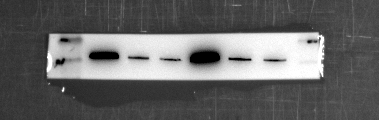


NSUN2


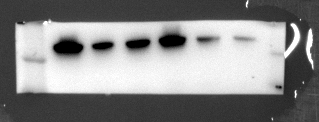


ARG-1


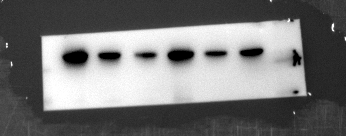


IL-10


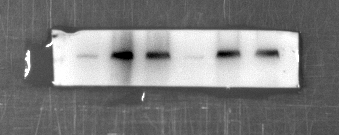


TNF-α


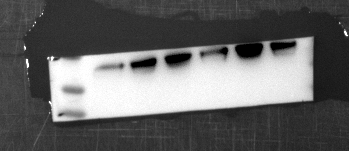


iNOS


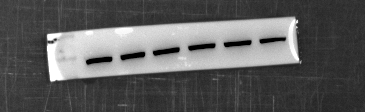


GAP

Figure2---A (THP-1+PBMC)


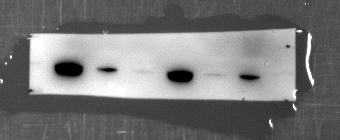


NSUN2


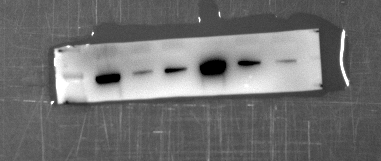


ARG-1


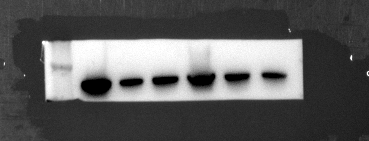


IL-10


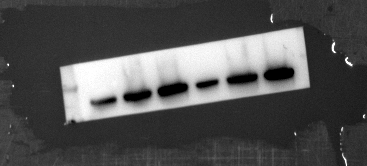


TNF-α


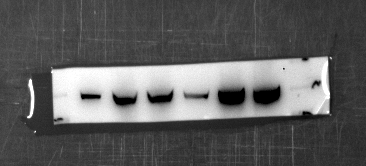


Inos


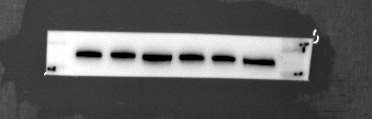
GAPDH

Figure2---D(RAW+BMDM)


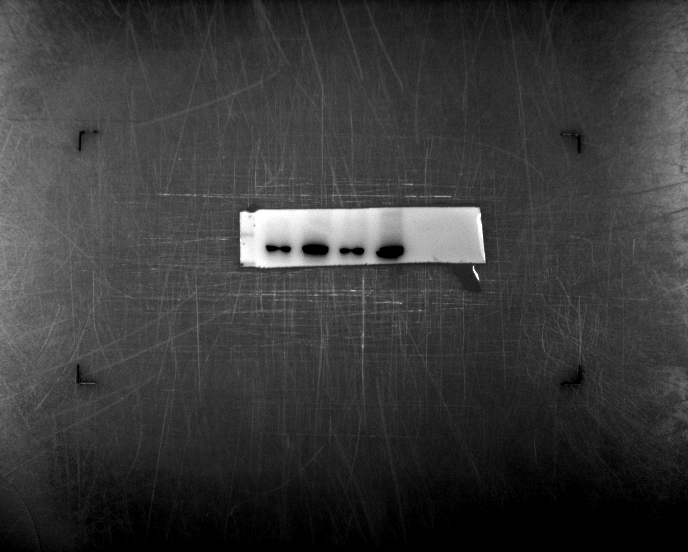


NSUN2


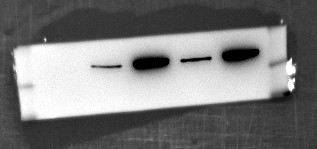


ARG-1


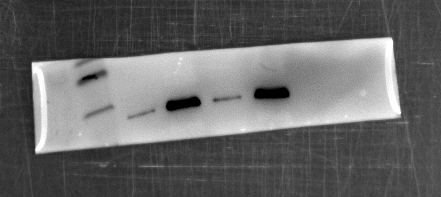


IL-10


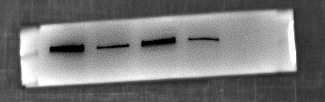


TNF-α


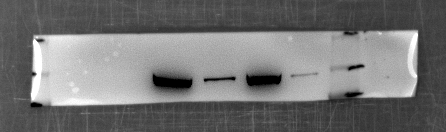


iNOS


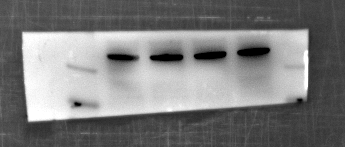


GAPDH

Figure2---D (THP-1+PBMC)


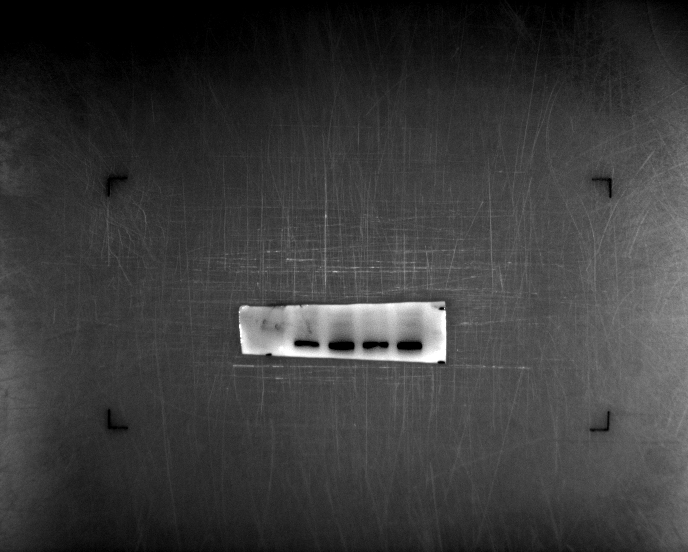


NSUN2


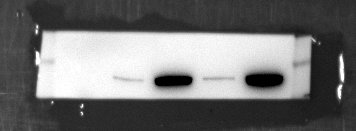


ARG-1


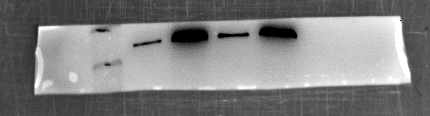


IL-10


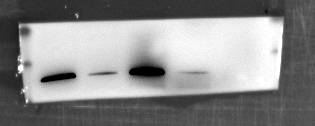


TNF-α


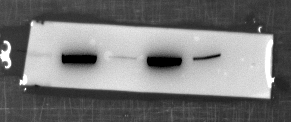


iNOS


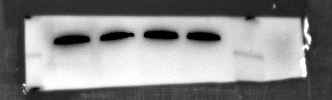


GAPDH

Figure3---D (RAW+BMDM)


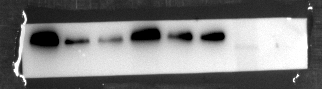


NSUN2


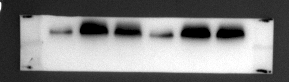


SOCS3


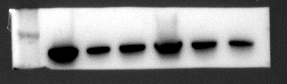


JAK2


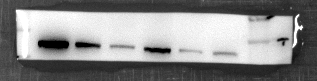


STAT3


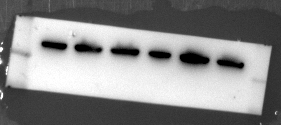


GAPDH

Figure3---D (THP-1+PBMC)


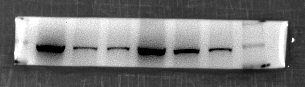


NSUN2


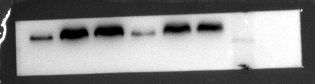


SOCS3


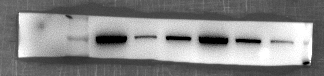


JAK2


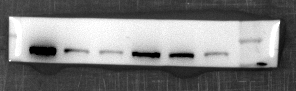


STAT3


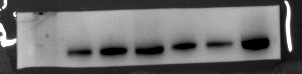


GAPDH

Figure4---A(RAW+BMDM)


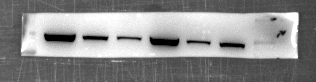


NSUN2


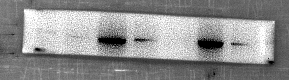


SOCS3


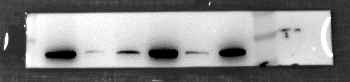


JAK2


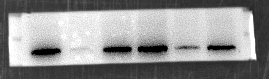


STAT3


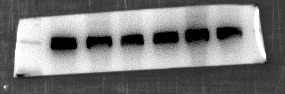


GAPDH

Figure4---A (THP-1+PBMC)


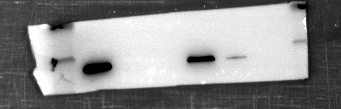


NSUN2


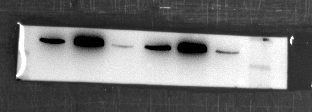


SOCS3


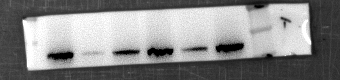


JAK2


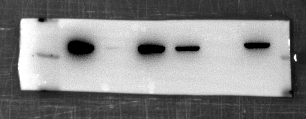


STAT3


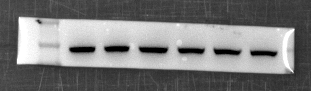


GAPDH

Figure4---C(RAW+BMDM)


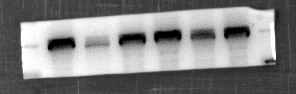


ARG-1


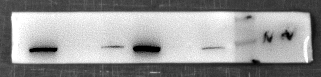


IL-10


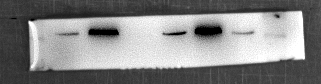


TNF-α


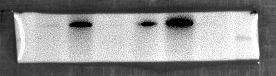


iNOS


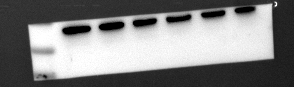


GAPDH

Figure4---C (THP-1+PBMC)


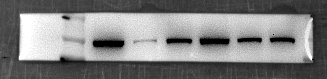


ARG-1


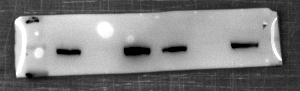


IL-10


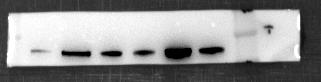


TNF-α


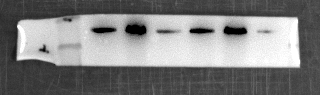


Inos


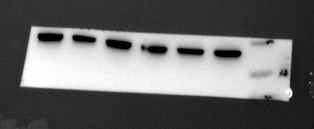


GAPDH

Figure5---A


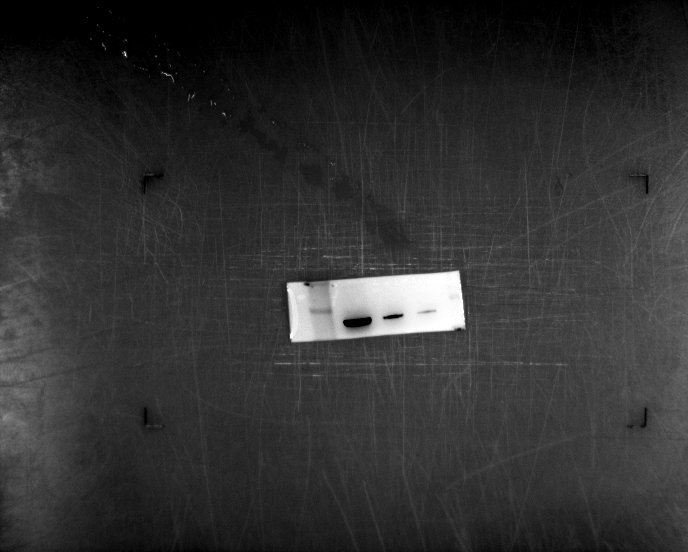


NSUN2


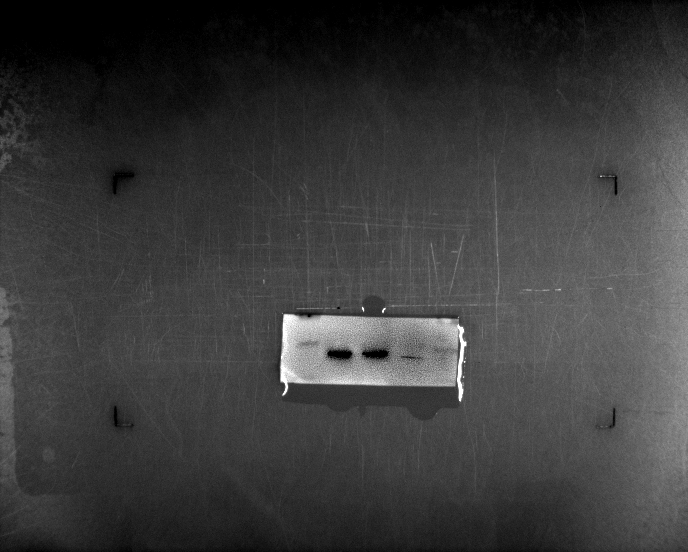


SOCS3


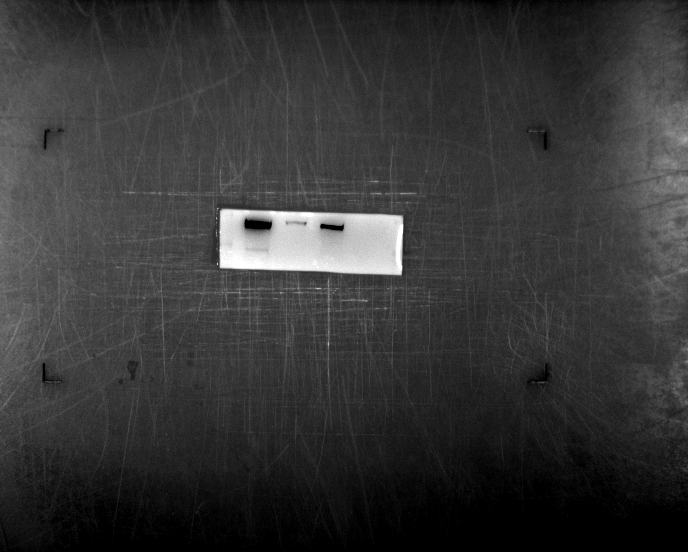


ARG-1


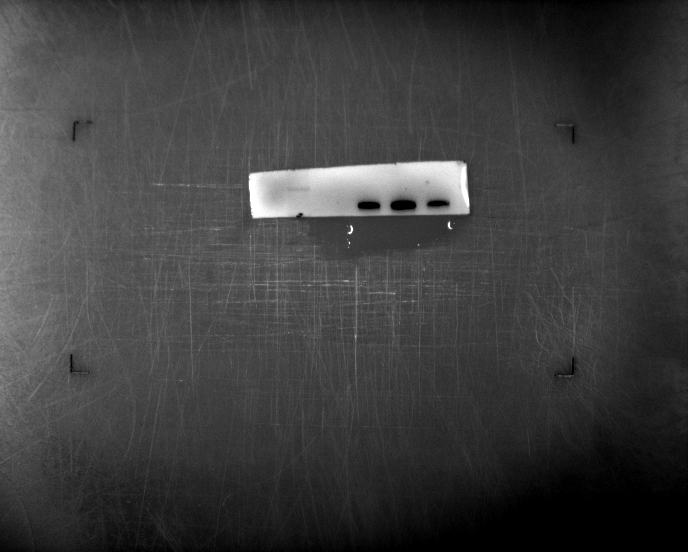


TNF-α


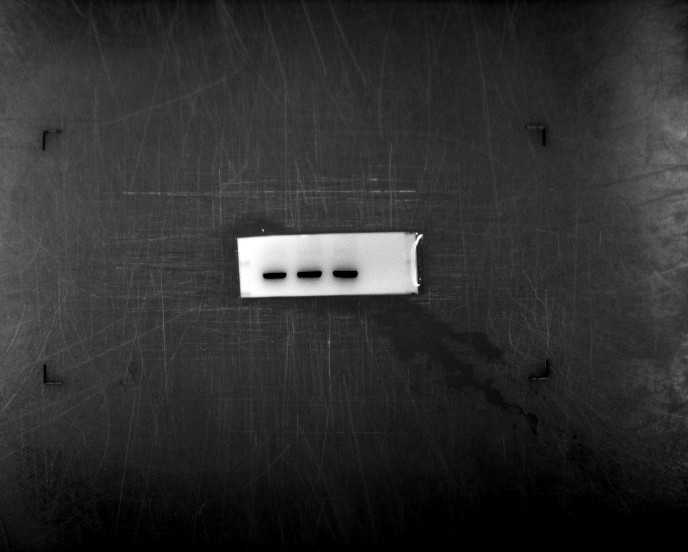


GAP

Figure7---D


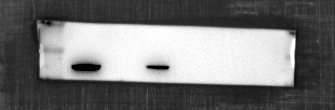


THP-1


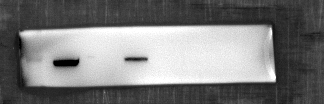


PBMC

Figure8---A


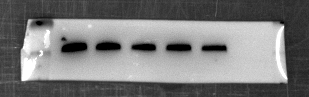


site1-input


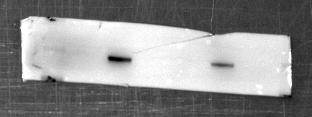


site1-output

Figure8---D-- siYBX1 group (RAW+BMDM)


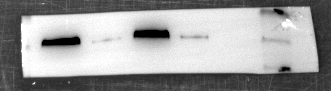


YBX1


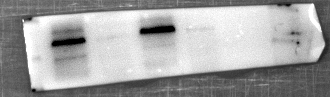


ARG-1


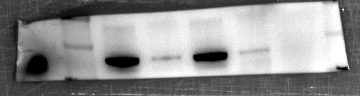


IL-10


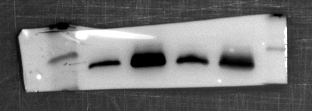


TNF-α


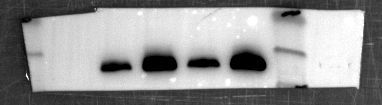


Inos


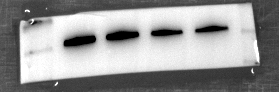


GAPDH

Figure8---D-- siYBX1 group (THP-1+PBMC)


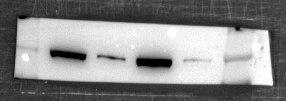


YBX1


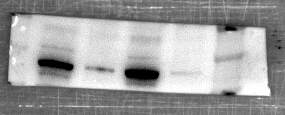


ARG-1


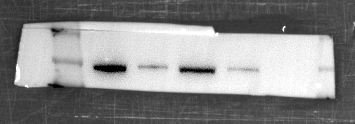


IL-10


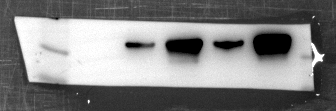


TNF-α


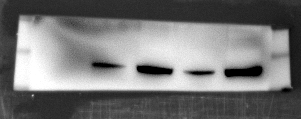


iNOS


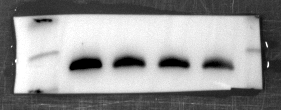


GAPDH

Figure8---D-- siTET2 group (RAW+BMDM)


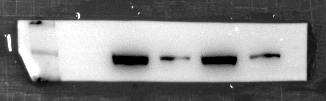


TET2


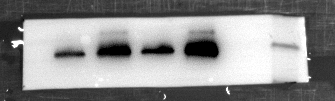


ARG-1


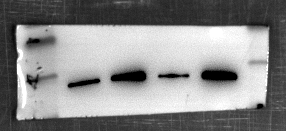


IL-10


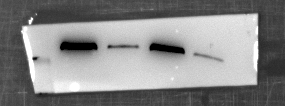


TNF-α


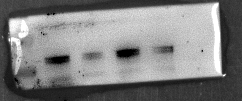


INOS


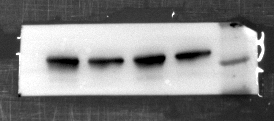


GAPDH

Figure8---D-- siTET2 group (THP-1+PBMC)


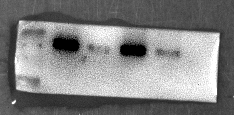


TET2


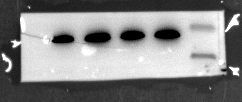


ARG-1


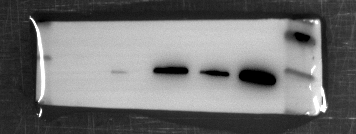


IL-10


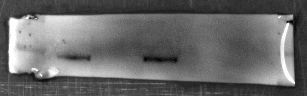


TNF-α


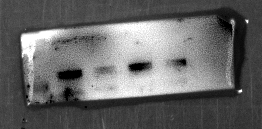


INOS


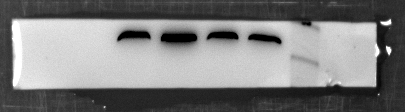


GAPDH

FigureS3---G-- M1 polarized group


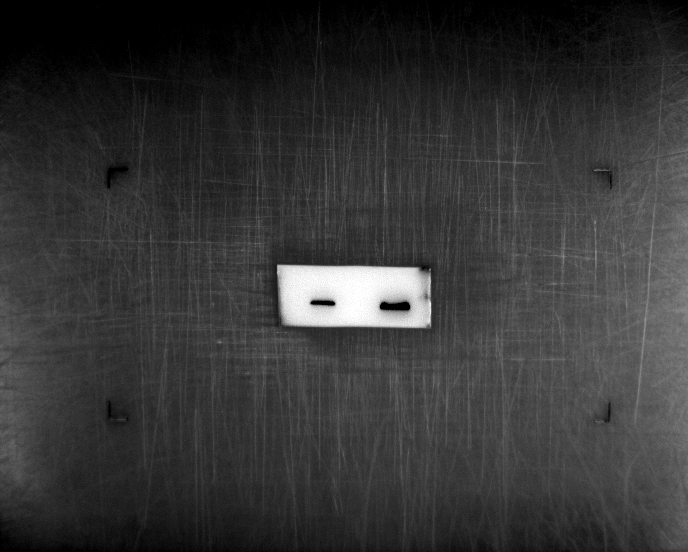


NSUN2


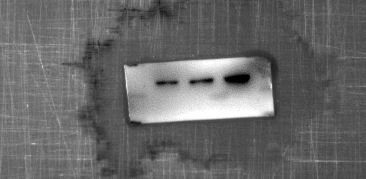


ARG-1


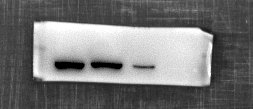


TNF-α


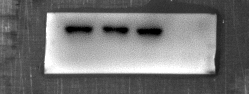


GAP

FigureS3---G-- M2 polarized group


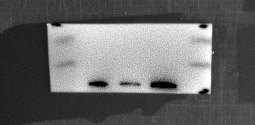


NSUN2


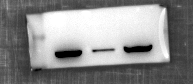


ARG-1


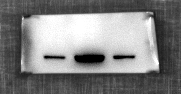


TNF-α


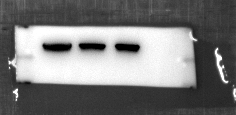


GAPDH
